# SAD: semi-supervised automatic detection of BOLD activations in high temporal resolution fMRI data

**DOI:** 10.1007/s10334-024-01197-0

**Published:** 2024-08-29

**Authors:** Tim Schmidt, Zoltán Nagy

**Affiliations:** 1 Laboratory for Social and Neural Systems Research, SNS–Lab, University of Zurich, Rämistrasse 100, CH-8091 Zurich, Switzerland; 2 Institute for Biomedical Engineering, ETH Zurich and University of Zurich, Zurich, Switzerland

**Keywords:** fMRI, BOLD, Autocorrelation, Neural networks, LSTM

## Abstract

**Objective:**

Despite the prevalent use of the general linear model (GLM) in fMRI data analysis, assuming a pre-defined hemodynamic response function (HRF) for all voxels can lead to reduced reliability and may distort the inferences derived from it. To overcome the necessity of presuming a specific model for the hemodynamic response, we introduce a semi-supervised automatic detection (SAD) method.

**Materials and methods:**

The proposed SAD method employs a Bi-LSTM neural network to classify high temporal resolution fMRI data. Network training utilized an fMRI dataset with 75-ms temporal resolution in an iterative scheme. Classification performance was evaluated on a second fMRI dataset from the same participant, collected on a different day. Comparative analysis with the standard GLM approach was conducted to evaluate the cooperative effectiveness of the SAD method.

**Results:**

The SAD method performed well based on the classification scores: true-positive rate = 0.961, area under the receiver operating curve = 0.998, true-negative rate = 0.99, F1-score = 0.979, False-negative rate = 0.038, false-discovery rate = 0.002, false-positive rate = 0.002 at 75-ms temporal resolution.

**Conclusion:**

SAD can detect hemodynamic responses at 75-ms temporal resolution without relying on a specific shape of an HRF. Future work could expand the use cases to include more participants and different fMRI paradigms.

**Supplementary Information:**

The online version contains supplementary material available at 10.1007/s10334-024-01197-0.

## Introduction

Functional Magnetic Resonance Imaging (fMRI) plays a key role in research into the human brain by allowing us to investigate behavior and cognitive functions non-invasively based on the coupling between neural activity and local cerebral blood oxygenation [1–3]. The blood oxygen level-dependent (BOLD) signal is the primary fMRI contrast mechanism, which is based on the different magnetic properties of arterial and venous blood [4–8].

The central aim of BOLD fMRI is to identify regions or networks of the brain that exhibit a detectable change in the MRI signal, i.e., above the present noise level, due to neurovascular responses originating from neuronal activation [9, 10]. However, a reliable and reproducible identification of BOLD activations entails a range of challenges, encompassing practical and theoretical aspects [11–16].

The conventional approach for finding activated brain regions in BOLD fMRI is based on fitting an assumed hemodynamic response function (HRF) to the data in a generalized linear model (GLM) framework [17]. The HRF represents a mathematical model of the hemodynamic response (HDR) to neuronal activity, which is assumed to be a linear time-invariant (LTI) system [17]. However, there is mounting evidence that the HDR varies substantially across brain regions, fMRI sessions, experimental conditions/stimuli, etc., leading to potential mismodelling and thus diminished reliability of the statistical analysis [11, 18–22]. The advances of parallel imaging [23–25] or fMRI data acquisition and reordering methods [26, 27] further exacerbate this problem, because sampling the BOLD response faster (i.e., with TR in the 10’s or 100’s of milliseconds) highlights more reliably when the assumed model does not fit the data—especially in experiments that impart small changes in the amplitude or timing of the otherwise sluggish BOLD response [22].

Although, various research endeavors have delved into the application of machine learning techniques [28] to fMRI [29] for the purpose of identifying networks of brain activity as well as for diagnosing and categorizing cognitive disorders [30, 31], less has been done on employing these methods at the first level—to identify voxels where a BOLD activation has occurred. In the present work, we develop and test a novel semi-supervised automatic detection (SAD) method for identifying voxel-wise BOLD activations in high-temporal resolution fMRI time-series data [26, 27]. The SAD method is model free (i.e., does not require an assumed HRF) and is based on a bi-directional long-short term memory (Bi-LSTM) neural network [32]. Previously, LSTM neural networks have been utilized for identifying cognitive disorders [31] and decoding brain states in fMRI [33]. After training the model, we assess its performance on an independent data set and compare its results to the conventional GLM-based analysis.

## Materials and methods

### The proposed SAD method

Let ${\overrightarrow{x}}_{v}$ be a time-series of voxel $v$, which samples the fMRI signal across ~ 20 s (i.e., the length of a BOLD activation in response to a brief stimulus or a quick motor action). The length, $T$, of the time-series is related to the temporal resolution (i.e., TR of the experiment). Under the assumption that an arbitrary voxel time-series $\vec{x}_{v}$ either belongs to the “activated” (class 1, $C_{1}$) or “non-activated” (class 0, $C_{0}$) class, we may define the fMRI dataset, H, to be a union of two mutually exclusive classes,

1 $$H = C_{0} \cup C_{1}$$

where $C_{0} = \left\{ {\vec{x}_{v}\,\, \textit{is not activated},v \in H} \right\}$ and $C_{1} = \left\{ {\vec{x}_{v}\, \textit{is activated},v \in H} \right\}$. The goal is to find a decision function that reliably separates voxels in an fMRI data set into these two classes.

Classification problems are typically supervised—they rely on data with ground-truth labels for training the parameters of the decision function. However, labelling a common fMRI dataset that may include 10’s of thousands of voxels is prohibitively time consuming. Thus, we proceed with a semi-supervised approach, where the initial labelling is automatic and data-driven, while the subsequent training is iteratively improved without supervision.

### Initialization (initial label assignments)

**Fig. 1:**
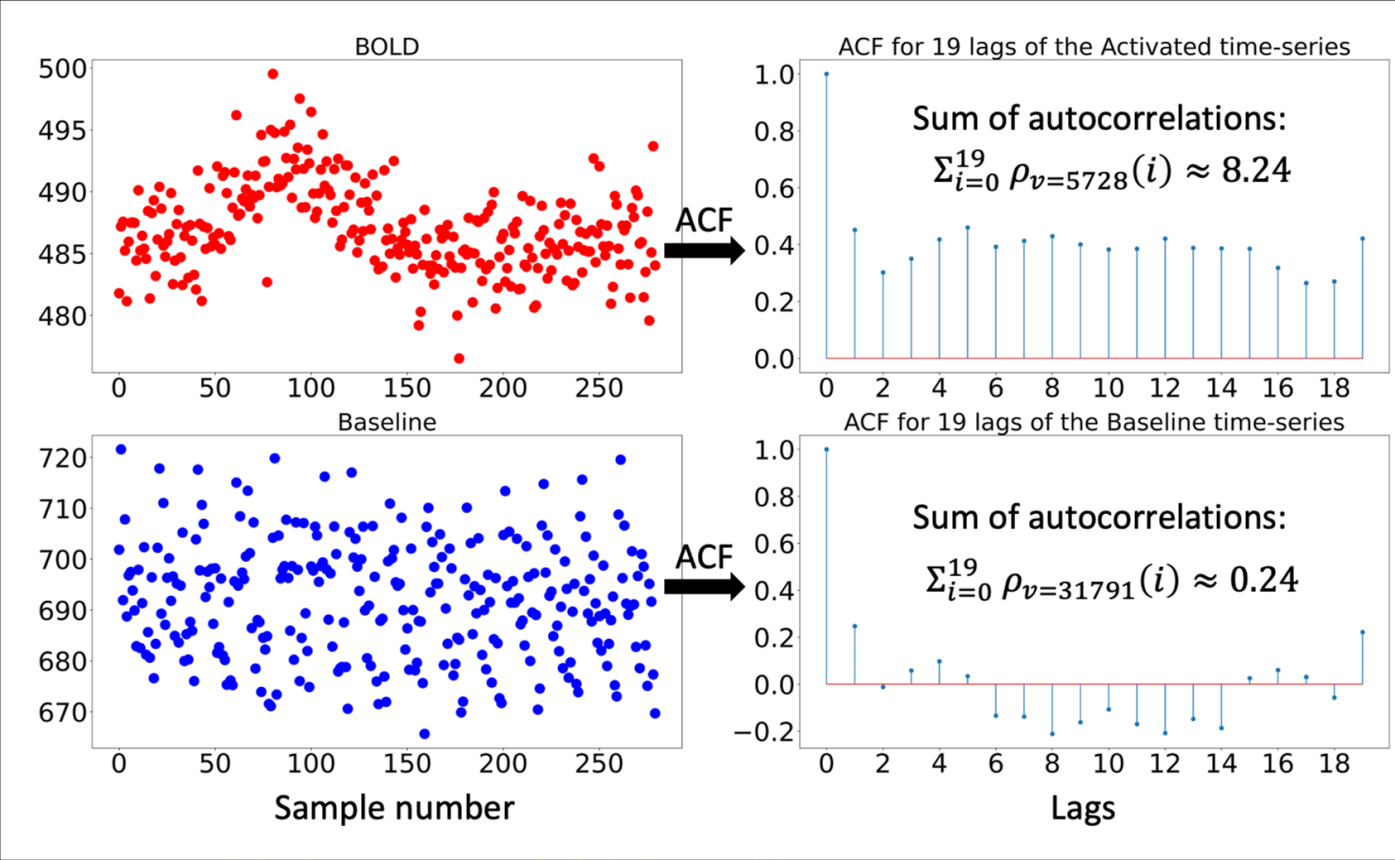
Visualization of the automatic and data-driven initialization procedure. Top row: display of the time-series of a voxel featuring a BOLD activation on the left and its computed autocorrelation function on the right. The autocorrelation function was evaluated at 20 time-lags, including the 0th term (always 1), yielding a total sum of the autocorrelations (${SA}_{v}$) of 8.24 for voxel 5728. Bottom row: similar plots and calculations but for voxel 31791 with a baseline time-series, yielding a ${SA}_{v}$ that is near 1 (0.24) as expected for a truly random signal. Note that on the *y*-axis of the sampled time-series are arbitrary intensity units, whereas on the *x*-axis are sample numbers of the time-series. *ACF* = autocorrelation function

One obvious difference between the time-series of voxels with and without a BOLD activation is that, while the baseline signal is more or less random, the BOLD signal evolution has a temporal structure (Fig. 1). Therefore, the autocorrelation of the time-series in voxels with a BOLD activation is different from that of voxels with the random background signal. We suggest using this difference to initially label a few voxels by computing the sample autocorrelation [34] of each voxel time-series, ${\overrightarrow{x}}_{v}$, repeatedly with increasing lag, and summing the sample autocorrelation [36] across all the lags

2 $$SA_{v} = \mathop \sum \limits_{i = 0}^{{h_{\max } }} \hat{\rho }\left( i \right)_{v}$$

where

3 $$\hat{\rho }\left( i \right)_{v} = \frac{1}{{Var\left( {\vec{x}_{v} } \right)\cdot\left( {T - i} \right)}}\mathop \sum \limits_{k = 0}^{T - i} \left( {x_{v} \left[ {k + i} \right] - \overline{x}_{v} } \right)\left( {x_{v} \left[ k \right] - \overline{x}_{v} } \right)$$

and $Var\left({\overrightarrow{x}}_{v}\right)$ and ${h}_{max}$ refer to the temporal variance of ${\overrightarrow{x}}_{v}$ and the maximal number of computed time-lags, respectively. ${h}_{max}$ is one of the hyperparameters of the network – here set to 19. Longer lags may be used if needed, but care should be taken that the autocorrelation function remains reliable. An example of the autocorrelation function and its sum across 19 lags are provided in Fig. 1 for two different voxel time-series—one with a clear BOLD activation and another with a random baseline signal.

**Fig. 2:**
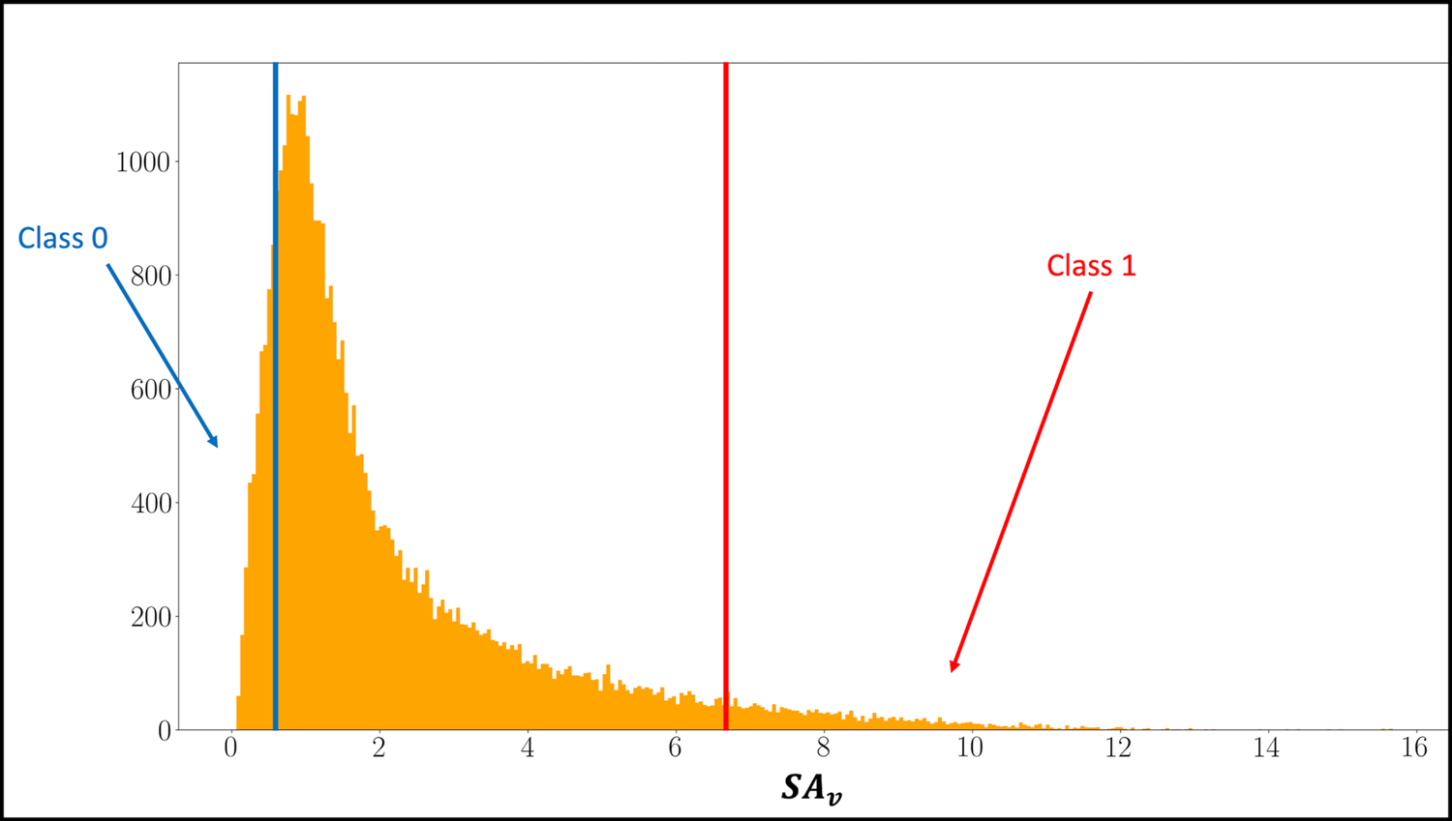
Initial label assignment. The histogram represents the empirical distribution of ${SA}_{v}$ (i.e., sum of autocorrelation of voxel time-series across the computed lags). ${SA}_{v}$ for each voxel, v, is computed using Eqs. (2) and (3). The blue demarcation line serves as the upper threshold for classifying voxel time-series as belonging to ${C}_{0}$. Similarly, a red demarcation line is the lower threshold for the value of ${SA}_{v}$ above which a voxel time-series is classified as part of ${C}_{1}$. The first training pool consists of the same number of samples in each class. A training sample is a combination of fMRI time-series and its automatically assigned label, i.e., $\left( {\vec{x}_{v} ,label} \right).$

The initial class labels 0 and 1 are assigned according to the lowest and highest $\frac{{\alpha_{initialization} }}{2}{-}quantiles \, of \, SA = \left\{ {SA_{v} } \right\}_{v = 1, \ldots ,\# voxels}$, respectively. For example, if ${\alpha }_{initialization}$ is 10% then voxels with $S{A}_{v}>quantil{e}_{0.95}(SA)$ and $S{A}_{v}<quantil{e}_{0.05}(SA)$ are labelled 1 or 0, respectively (Fig. 2).

${\alpha }_{initialization}$ is another hyperparameter and is computed according to an optimization problem

4 $$\arg \min_{{\alpha \in \left[ {0,1} \right]}} \left\{ {\left( {E\left[ {SA | SA \ge q_{{1 - \frac{\alpha }{2}}} \left( {SA} \right)} \right] - E\left[ {SA | SA \le q_{{\frac{\alpha }{2}}} \left( {SA} \right)} \right]} \right) \cdot \frac{1}{{\max \left\{ {SA} \right\} - \min \left\{ {SA} \right\}}} + \alpha } \right\},$$

where the two summands are decreasing and increasing with $\alpha$, respectively, and thus provide a trade-off between the number of training samples and the difference of mean $SA$ among the voxels belonging to the sets of the two classes, ${C}_{1}$ and ${C}_{0}$. In other words, we desire a large initial training pool, in which the time-series belonging to the two classes differ as much as possible in their temporal autocorrelation structure. Note that $E\left[SA | SA\ge {q}_{1-\frac{\alpha }{2}}\left(SA\right)\right]$ is also known as the tail-value-at-risk, a quantity often used in extreme value theory [35]. The voxels thus labelled will be called the *initialization pool* and are used in the first iteration of training the neural network.

#### Iterative neural network training

**Fig. 3:**
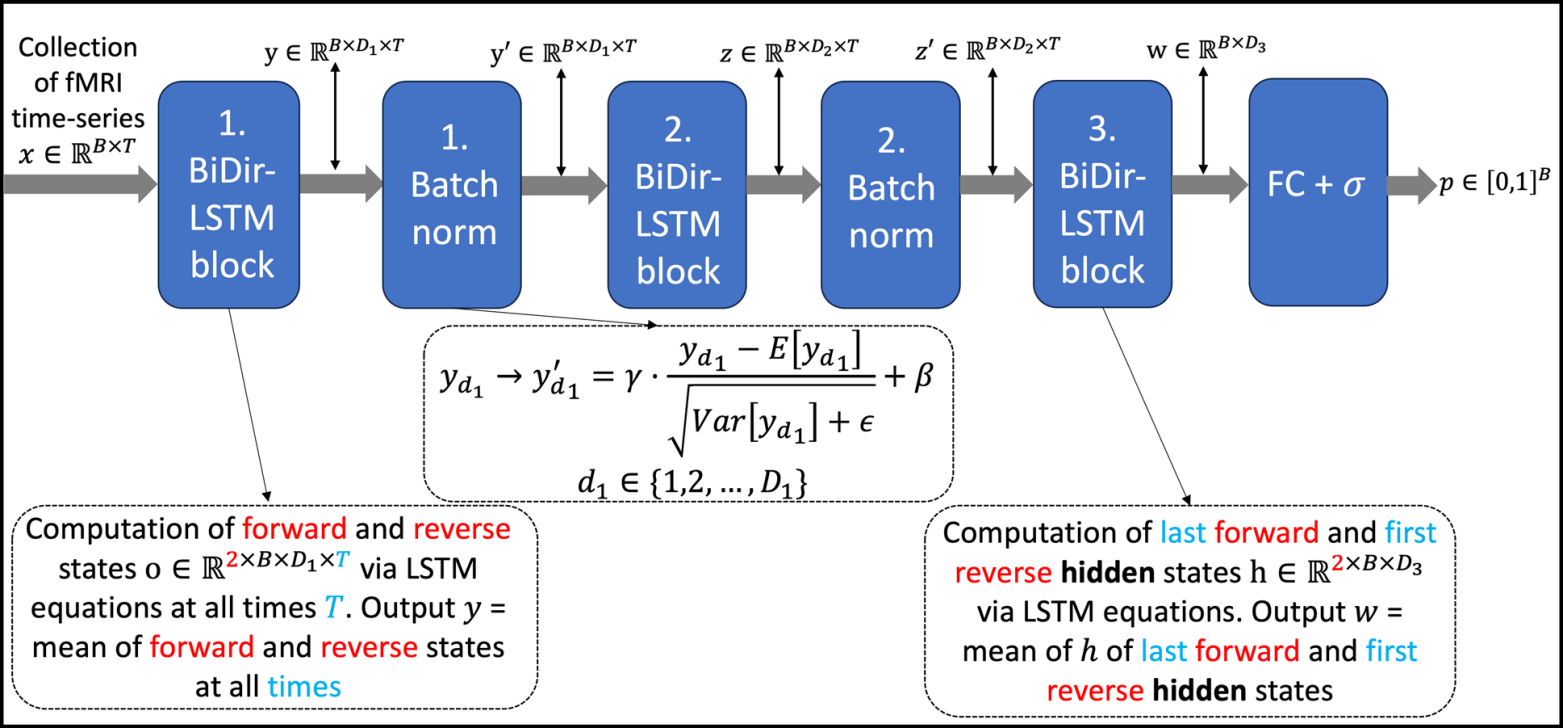
Neural network structure. To begin, a batch of size of $B$ fMRI time-series of length $T$ are fed into the first Bi-LSTM [32] block. Via the LSTM equations, forward and reverse states denoted as $o$ of size ${D}_{1}$ (first hidden dimensions) are computed at all times, $T$. The subsequent output, denoted here as $y$, of the Bi-LSTM block is then the mean of the computed forward and reverse states. Next, the output $y$ undergoes a batch normalization [36] (output $y{\prime}$) before it is entered into the second Bi-LSTM block with hidden dimension ${D}_{2}$ (ouput $z$). In the last Bi-LSTM block (output $w$), the last forward and first reverse hidden states are averaged out and enter a fully connected layer, FC, with sigmoid activation function (denoted as $\sigma$), which finally maps the output of each sample in the batch into the interval $[\text{0,1}]$, resulting in vector $p$ of size $B$ with values in $[\text{0,1}]$

**Fig. 4:**
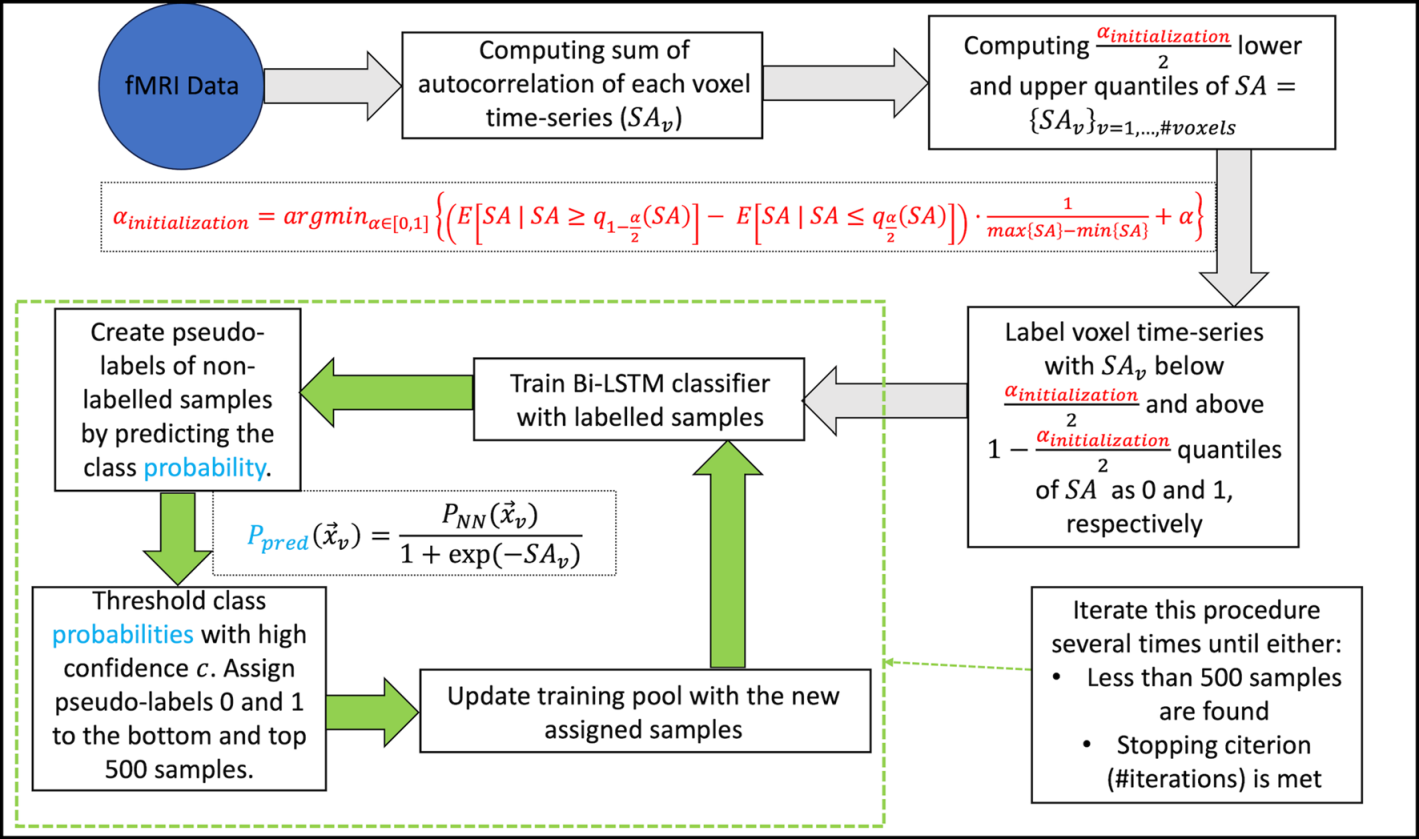
Flowchart of SAD. Initially, we compute the sum of autocorrelation of each voxel time-series ($S{A}_{v}$) across 20 time-lags (see also Fig. 1 and 2). Subsequently, using Eq. (4) (highlighted in red), ${\alpha }_{initialization}$ is computed and voxel time-series, ${\overrightarrow{x}}_{v}$, with $S{A}_{v}$ below $\frac{{\alpha }_{initialization}}{2}$ and above $1-\frac{{\alpha }_{initialization}}{2}$ are assigned with label 0 and 1, respectively to create the initialization pool. After first training the neural network with the initialization pool, class probabilities are predicted via ${P}_{pred}\left({\overrightarrow{x}}_{v}\right)= \frac{{P}_{NN}({\overrightarrow{x}}_{v})}{1+exp(-S{A}_{v})}$ for all unseen voxels and thresholded with high confidence level $c$. Pseudo-labels 0 and 1 are assigned to the bottom and top 500 samples. For example, a sample is assigned with label 1 if ${P}_{pred}>c$ and it is entered into the set if it has one of the 500 highest ${P}_{pred}$. Finally, the training pool and the neural network are updated with the newly assigned samples. The updating procedure is stopped when either less than 500 samples are found or another stopping criterion such as the total number of iterations is met. At each iteration the newly added samples are split 70% vs 30% into further training and validation sets, respectively

After the initialization, a neural network (Fig. 3) that consists Bi-LSTM [32, 36] blocks, batch normalization [36] and a fully connected layer with sigmoid activation functions is trained by minimizing the cross-entropy loss in an iterative scheme (Fig. 4):

1. At the first iteration, the neural network is trained with the automatically labelled initialization pool.
2. After the first training period, pseudo-labels [37] are created for all voxels that did not belong to the initialization pool with the model trained in Step 1. To identify and use only the most confident predictions, a voxel $v$ is assigned with label 0 and 1 if ${P}_{pred}\left({\overrightarrow{x}}_{v}\right)<1-c$ and ${P}_{pred}\left({\overrightarrow{x}}_{v}\right)>c$, respectively, where $c$ is a hyperparameter called the confidence level that was set to 0.98. At each iteration, the 500 voxels with the most extreme ${P}_{pred}$ in each class are added to the initialization pool, thereby creating the expanded training pool for the next iteration. The function ${P}_{pred}(\cdot )$ consists of the network output, ${P}_{NN}\left(\cdot \right)$ (see Fig. 4) times an additional function based on $S{A}_{v}$: ${P}_{pred}\left({\overrightarrow{x}}_{v}\right):= \frac{{P}_{NN}({\overrightarrow{x}}_{v})}{1+\text{exp}(-S{A}_{v})}$. The denominator acts as a regulatory factor, increasing the difficulty for samples in subsequent iterations to be categorized into $C_{1}$, which helps reduce the class imbalance that would otherwise occur because in any given fMRI experiment voxels without a BOLD activation outnumber those with an activation.
3. Repeat step 2 and keep adding newly labelled voxels into the training pool unless the new iteration adds less than 500 $C_{1}$ labels or a prior set number of iterations is reached.

Step 2 requires choosing the hyperparameter $c$, defined by

5 $$- \log \left( {\frac{1}{c} - 1} \right) \le quantile_{{1 - \frac{{\alpha_{initialization} }}{2}}} \left( {SA} \right)$$

where $\text{log}(\cdot )$ denotes the natural logarithm and, similarly to ${\alpha }_{initialization}$ above, ensures that only voxels whose labels can be assigned confidently will be added to the training pool. Like setting hyperparameters in general, determining the optimal confidence level is an empirical problem. We have settled on $c=0.98$ which fulfills Eq. (5) and provided reliable results.

At each iteration, the neural network is trained using the Adam optimizer [38], batch size of 32, 50 epochs, 0.001 learning rate and hidden dimensions of 15, 10 and 3 (correspond to ${D}_{1},{D}_{2}$ and ${D}_{3}$ in Fig. 3). In total, the network has 4734 parameters.

Additionally, in each iteration, the sample pool undergoes a pseudo-random split, with 70% allocated to the training set and 30% to the validation set, where cross-entropy losses are then computed.

The neural network is programmed in Python (version 3.9.12) with PyTorch (version 2.0.1).

After the neural network is trained, class probabilities are predicted using the trained model across either all voxel time-series of the same data set or approximately 12,000 manually labelled voxels of an independent 2nd data set (further details below).

### Motor and visual task

The stimulus paradigm was previously reported in Schmidt et al. [26]. Briefly, the visual stimuli were created using nordicAktiva (Nordic NeuroLab, Bergen, Norway) software and presented on an MRI-compatible screen measuring 40 inches in diameter with a resolution of 3840 × 2160 pixels. The participant was instructed to focus on a small white cross situated at the center of a uniform gray (R/G/B-127/127/127) background.

The visual stimulus was a flickering (8 Hz for 375-ms) black and white checkerboard whose appearance also served as a cue for the participant to squeeze a response grip with the dominant hand. These motor responses were slightly delayed relative to the visual stimulus.

The paradigm encompassed 20 visual stimuli, which were slightly and progressively shifted relative to the acquisition repetition time (TR) to coincide with the time of acquisition of one of the 20 slices on consecutive trials. The 20 stimuli were repeated 5 times, but pseudo randomly on one of the 5 repetitions the visual stimulus (and hence the motor response) was omitted for each of the 20 slices.

### Data acquisition

#### Participants

The Cantonal Ethics Committee of Zurich (2020-00208_TS12) approved the study, which involved one right-handed male adult participant, who signed a written informed consents prior to scanning. The participant was scanned twice on two different days with the same stimulus paradigm and imaging protocol. The first dataset was used for training and validation of the SAD method, while the second dataset was manually labelled and used as a test set for the trained model. Henceforth, the set of gray matter voxels extracted from the first fMRI dataset will be denoted as S_Train_, while that from the second data set will be denoted as S_Test_.

#### Imaging protocol

The imaging protocol was previously reported in Schmidt et al. [26]. Briefly, the data were collected on a 7T MAGNETOM Terra scanner (Siemens Healthineers, Erlangen, Germany) equipped with a single-channel transmit and 32-channel receive head coil (Nova Medical Inc., MA, United States). The acquisition utilized a 2D EPI sequence [39] with a multiband acceleration factor of 3 [23, 25] to collect 60 slices and provide full brain coverage. Further sequence parameters can be found in Table 1. Additionally, a 3D T1-weighted MP2RAGE [40] image with 1-mm isotropic resolution was collected for anatomical guidance and extracting the gray matter segment from the EPI images.

**Table 1:** Acquisition sequence parameters

Sequence parameters	
TR [ms]	1500
TE [ms]	25
Flip angle [°]	73
Voxel size [mm^3^]	2 × 2 × 2
EPI volumes	1410
Bandwidth/pixel [Hz/Px]	2066
Slice orientation	Transversal
Phase encoding direction	Anterior/posterior
Partial fourier	6/8
Acquisition time [min:s]	35:25

## Data processing

### HiHi in a nutshell

**Hi**gh temporal resolution and **hi**gh signal-to-noise ratio (HiHi) fMRI utilizes a data reordering approach. By selecting a prolonged TR, thereby facilitating more complete T1 relaxation and enhancing the signal level, this technique achieves an effectively shortened TR through strategic reshuffling of the initial slices and thus a high effective temporal resolution of the BOLD signal. The stimulus timing is adjusted relative to the EPI acquisition by shifting it for each repeat to match RF excitation of a different slice (see Supplementary Fig. 4). The slice where a stimulus occurs gets mapped to the same slice of the first HiHi time-point, while post-stimulus acquired slices retain their slice position but are placed in consecutive HiHi time-points. A particular voxel within the high temporal resolution (HiHi) time-series (highlighted in yellow in Supplementary Fig. 4), potentially indicating a BOLD activation, can be identified either manually or through the application of a detection algorithm such as SAD or model-based regression such as GLM.

### Pre-processing

Before any machine learning analysis and processing, the raw fMRI data were pre-processed using custom-made Matlab scripts (version 2021a and 2023b; The MathWorks, Inc., Natick, MA, USA) and SPM 12 (Wellcome Centre for Human Neuroimaging, UCL, London, England) [17]. The data from the four repeats where the visual stimulus was presented (and hence the motor response was performed) were the main interest in this study. The repeat where the stimulus was omitted served as an estimate of the baseline signal and used only for scaling the corresponding voxel-signal from the four repeats with the stimuli so the latter could be displayed in % units. Before the HiHi reshuffling step (for detailed description see [26, 27] and for a brief summary see the *HiHi in a Nutshell* subsection above) both the S_Train_ and the S_Test_ data were 3D rigid-body aligned to the first EPI volume of each run and subsequently each voxel time-series was detrended via a second-degree polynomial fit. The HiHi reshuffling was based on the timing of the visual response or the timing when the stimulus would have occurred but was randomly omitted to measure the baseline signal in each voxel. Both conditions resulted in a pseudo time-series of 21 s with 75 ms temporal resolution [26]. The motor responses were delayed by approximately half a second on average. This presented no issue for SAD, but to address this time shift in a GLM approach, we incorporated the temporal derivative of the canonical HRF in one of the models’ design matrices.

The voxel time-series of both the S_Train_ and the S_Test_ data were standardized to zero mean and a standard deviation of 1 before entering the neural network. Finally, a gray matter mask was computed by segmenting the anatomical T1 image and thresholding the gray matter segment at 0.7 using SPM. The mask was then used to extract the gray matter voxel intensities from the EPI images of both S_Train_ and S_Test_.

### Data analysis

Data analysis was performed with custom-made Matlab and Python scripts (version 3.9.12).

#### Testing robustness of method on a test dataset

**Fig. 5:**
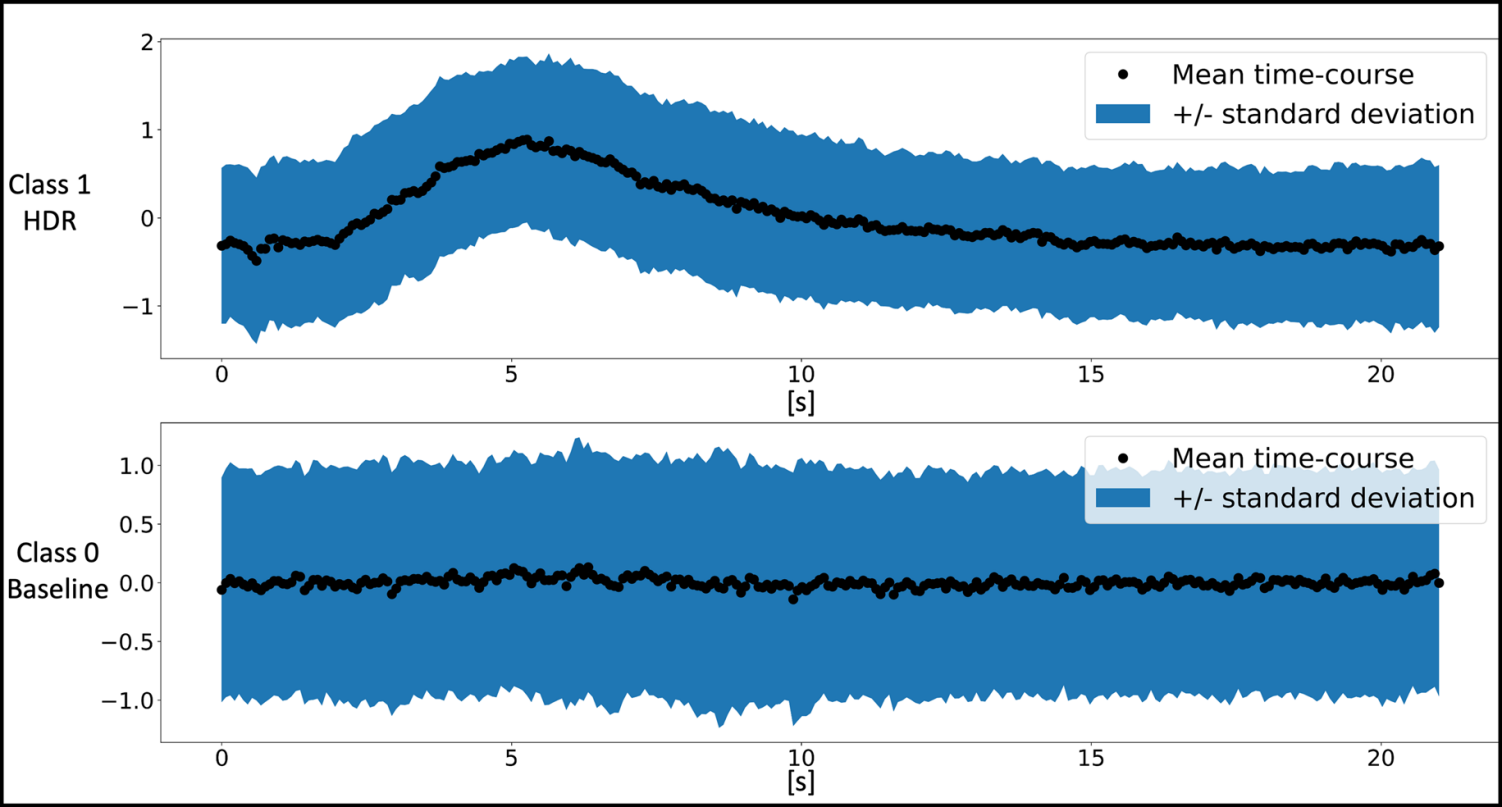
Test set mean time-courses with standard deviation bounds. Top row: the black dots illustrate the mean, while the blue shaded area represents ± 1 standard deviation, computed at each time point across the manually labeled ~ 6000 time-series for $C_{1}$ in S_Test_. Each time-series was individually centered (zero-mean) and normalized (standard deviation of 1) before mean and standard deviation were computed at each time point. Bottom row: same kind of plot but for the ~ 6000 time-series belonging to $C_{0}$ in S_Test_

As mentioned in the *Iterative neural network training* section above, the S_Train_ data were used as a training and validation set. Although the SAD method does not require manual labelling of the individual voxel time-series and is in that sense “unsupervised”, ascertaining the reliability of its performance can be carried out on independent and manually labelled data: the S_Test_ dataset from the same participant that was acquired with the identical stimulus paradigm and put through the same pre-processing steps was used for this purpose. The two authors independently and manually assigned labels to 12,000 voxel time-series from S_Test_. When the two authors could not agree on the appropriate label, or they agreed that voxel time-series was too ambiguous to assign a label with confidence, the voxel was excluded from the test set. This exclusion constituted 1.3% of the 12,000 voxels examined. The mean time-course across the manually labelled voxel time-series including the standard deviation bounds are displayed for both classes in Fig. 5.

To evaluate classification performance, 500 time-series were sampled from the approximately 6000 labelled voxels in each class and classified by the trained model. Classification performance was assessed by the area under the ROC (receiver operating characteristic) curve (AUC) (*roc_auc_score* function from sklearn version 1.0.2), as well as True Positive Rate (TPR), True-negative rate (TNR), positive-predictive value (PPV), Accuracy (ACC), F1-score, False-negative rate (FNR), False-positive rate (FPR), False-discovery Rate (FDR) and False-omission rate (FOR). This classification and performance evaluation was repeated 100 times on different random samples of 500 out of ~6000 labelled time-series in both classes and the mean and standard deviation of the performance scores were calculated across the 100 repeats.

#### Comparison GLM vs. SAD

Involving the ~12′000 labelled voxels from the S_Test_ data, three different HRFs were considered. Since most of the scanner related drifts were already regressed out in the HiHi fMRI time-series prior to the reshuffling step, the following simple GLM was considered first.

6 $$Y = \left( {h_{1} ,k} \right) \cdot \beta + \epsilon$$

Where ${h}_{1}\in {\mathbb{R}}^{T}$ is the canonical HRF from SPM and $k=\left(\text{1,1},\dots ,1\right)\in \mathbb{R}^T$. Next, the time and dispersion derivatives of ${h}_{1}$, denoted as ${h}_{2}$ and ${h}_{3}$, were included.

7 $$Y = \left( {h_{1} ,h_{2} ,h_{3} ,k} \right) \cdot \beta + \epsilon$$

Finally, the data were fit with a finite impulse response (FIR) model [15]

8 $$Y_{t} = \mathop \sum \limits_{k = 1}^{{n_{box} }} \beta_{k} \cdot \left( {\Theta \left( {t - \left( {k - 1} \right) \cdot w} \right) - \Theta \left( {t - k \cdot w} \right)} \right) + \beta_{{n_{box} + 1}} + \epsilon_{t}$$

where Θ (*x*) is the Heaviside step-function, which is 1 for $x\ge 0$ and 0 otherwise. We used 10 or 20 boxcar functions with a corresponding window length of approximately 2 and 1 s. The classical F-test, utilized for the design in Eq. (7), will be denoted as F_Class_, while those associated with Eq. (8) will be labeled as F_FIR10_ and F_FIR20_. The t-test belonging to Eq. (6) will be simply denoted as t.

After computing test statistics of the GLM, one needs to specify a threshold beyond which an activation is expected. In contrast to the SAD method, where the output ranges between [0,1] and values closer to 1 are considered as activations, the t and F test statistics are unbounded, and their thresholding is typically contingent on p-values. While we selected a 0.5 threshold for SAD, the thresholds for GLM test-statistics were derived from the family-wise error rate (FWER) at *p* = 0.05 in SPM [17].

As a comparison between both methods, we plot the predicted SAD outputs versus the GLM test-statistics in a scatter plot for both classes from S_Test_ together with their respective thresholds. Additionally, when there is a discrepancy (or consistency) between SAD prediction and GLM test-statistics on a notable portion of voxels, we display the mean time-course and its corresponding mean fitted response.

Finally, we show a spatial visualization of all voxels in S_Test_ predicted as active by SAD and/or GLM, including their overlay and mismatch in 3D rendered surface plots that were generated in FSL (version 6.0.7.10, http://fsl.fmrib.ox.ac.uk/fsl).

In a conventional fMRI experiment, only the data of interest are collected from each participant (i.e., no effort is made to collect separate training and testing sets). Therefore, the ultimate objective of the proposed SAD method is to classify all the voxels of the dataset from which a fraction of the voxels was used for training. Thus, in addition to classifying the labelled voxels in S_Test_, we also applied the trained network to all voxels in S_Train_. Representative BOLD time-series of S_Train_ together with their corresponding fitted GLM responses, SAD predictions and locations in the brain will be provided.

For both S_Train_ and S_Test_, the used model parameters of the Bi-LSTM NN are those which corresponded to the iteration number with the lowest validation loss in S_Train_ (not explicitly shown).

### Classification performance at different temporal resolutions

The SAD method presented above was tailored for the analysis of fMRI data with a temporal resolution of 75-ms, which was achievable by the HiHi reshuffling method [26, 27]. Because such high temporal resolution is not common, the generalizability of the method was assessed by temporally downsampling the original data. For each dataset with its own progressively lower temporal resolution, the sum of autocorrelation (see Eqs. (2–4)) was re-evaluated before training the SAD network. The hyperparameter ${h}_{max}$ in Eq. (2) was adapted by the following rationale: for a white noise time-series, the $S{A}_{v}$ is asymptotically normally distributed with variance proportional to $\frac{{h}_{max}}{T}$ [34], where $T$ is the length of the time-series. This assertion, while not be strictly true for time-series with a BOLD activation, functions as a heuristic to aid in the determination of an approximation in that a lower temporal resolution (i.e., decreasing T) will increase the variance of $S{A}_{v}$ also in a BOLD time-series. To counteract this increasing variance, we, therefore, set ${h}_{max}\to \lceil \frac{{h}_{max}}{d}\rceil$, for a given downsampling factor $d$ (e.g., for $d=2$, the temporal resolution is halved to 150 ms), where $\lceil \cdot \rceil$ is the ceiling function, i.e., it rounds up to the next integer. While this method mitigates the growing variance in $S{A}_{v}$ at lower temporal resolutions, the accuracy of initial label assignment may not be maintained as effectively as at 75-ms temporal resolution because the difference between $C_{1}$ and $C_{0}$ samples, estimated by $S{A}_{v}$, decreases with lower temporal resolution (see Supplementary Fig. 1a–e).

## Results

**Fig. 6:**
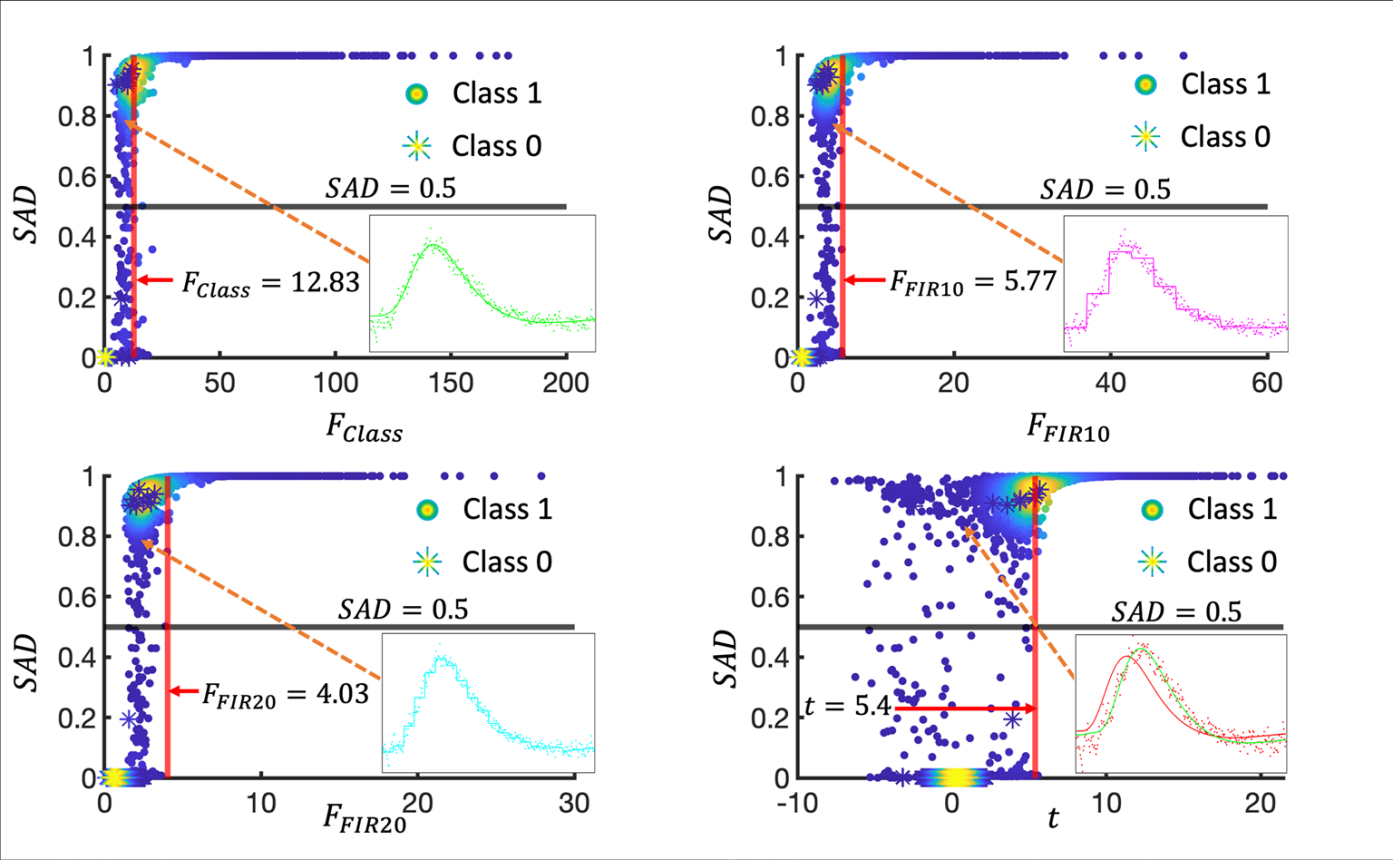
Scatter plot between SAD classification probabilities and F/t-statistics from S_Test_. Scatter plots between SAD probabilities and F_Class_ (upper left), F_FIR10_ (upper right), F_FIR20_ (bottom left) and t (bottom right) tests are given for the labelled $C_{1}$ (circles) and $C_{0}$ (asterisks) voxel time-series. The different colors of the circles and asterisks show their respective density in the scatter plot where brighter colors indicate a larger density of scatter points. The horizontal black and vertical red line indicate the activation thresholds for SAD (0.5) and the GLM test-statistics (*p* = 0.05 FWER), respectively. The mean time-courses, along with their mean fitted responses, are depicted in small insets within each scatter plot for the situations where SAD exceeds the 0.5 threshold, but GLM test-statistics are below their *p* = 0.05 FWER threshold. These time-series would be considered false positives from the GLM point of view, although the insets with the mean across these voxels indicate true positives. An orange dashed arrow is included solely as a visual guide to indicate the previously mentioned regions. All inset plots are scaled in [%] units and each mean response has an effect size of ~ 1%

Figure 6 provides scatter plots of SAD classification probabilities against t, F_class_, F_FIR10_, and F_FIR20_ statistic, for each of the labelled $C_{1}$ (circles) and $C_{0}$ (asterisks) voxel time-series that at least one of the methods identified as a BOLD activation in S_Test_ (~12′000 voxels in total). A substantial proportion of the time-series in the $C_{1}$ class are located in the region above the horizontal black and to the left of the vertical red demarcation lines. This indicates a disagreement between SAD and the other GLM test statistics—which may be considered true positives from the point of view of SAD or false positives from the point of view of the GLM statistics. The inset plots depict the corresponding mean time-course (dots) and mean fitted responses (solid lines) across these voxel time-series, suggesting a viable BOLD signal. The effect sizes of these four mean fitted responses are all ~ 1%. One notable result is the evident temporal disparity between the average fitted response depicted by the canonical HRF fit (solid red line) and the average voxel time-course (red dots) in the lower right graph. This temporal difference (~ 1.5 s) between the data and the assumed model is the likely reason for the t test failing to detect these BOLD activations. For comparison, the average fitted F_Class_ response for this set of voxels is also overlaid (solid green line). Note that the average time-courses shown in the four inset plots include a fraction (~ 2–5%) of responses with a negative t-value.

Conversely, 22 time-series of $C_{1}$ were placed below the 0.5 threshold of SAD but above the *p* = 0.05 FWER threshold of F_Class_ (dots below the black and to the right of the red demarcation lines), and hence could be considered false positives from the SAD point of view and true positives from the F_Class_ point of view. It was found that half of these time series had a negative activation pattern (i.e., negative *t* value), which is illustrated in Supplementary Fig. 2a (top row).

For completeness, the mean time-courses, where both SAD and GLM agree on the classification (either both 1 or both 0), or where SAD did not classify the time-course as 1 but GLM indicated an activation, are illustrated in Supplementary Fig. 3 along with their corresponding fitted responses.

**Fig. 7:**
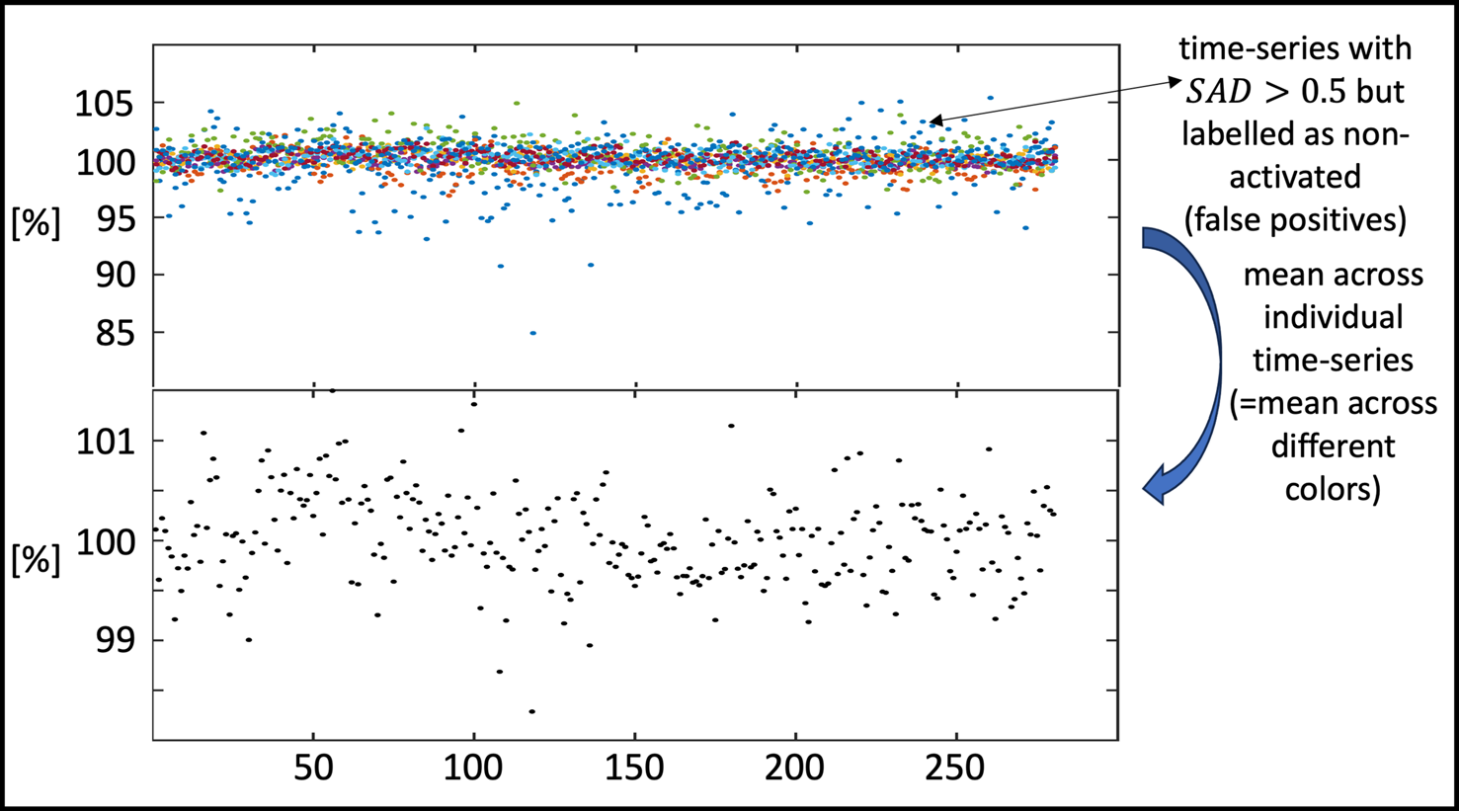
Time-series of the SAD false positive classifications from S_Test_. (upper row) Individual false-positive (i.e., SAD > 0.5) voxel time-series (different color for each time-series) from S_Test_. (bottom row) Mean time-course across the individual color-coded time-series in the upper row. Note that all time-series are scaled in [%] with respect to the mean time-course of the corresponding voxel in the baseline experiment (i.e., the repeat where the stimulus was omitted)

Except for the t-test, where it occurred for only two time-series, the GLM test statistics did not yield any false positives (i.e., asterisks are to the left of the *p* = 0.05 FWER thresholds) whereas SAD falsely assigned a value above the 0.5 threshold for 8 time-series from $C_{0}$. These can be considered false positives from the SAD point of view and true negatives from the GLM point of view. Figure 7 provides these time-series as individually colored dots (upper row) and their mean time-course (black dots, bottom row), which indeed shows a slight increase in signal intensity around the 50th time-point (~ 3.75 s). This slight increase, which is not easily discernible in the individual time series may be the reason why the SAD method classified these time-series as activations.

**Fig. 8:**
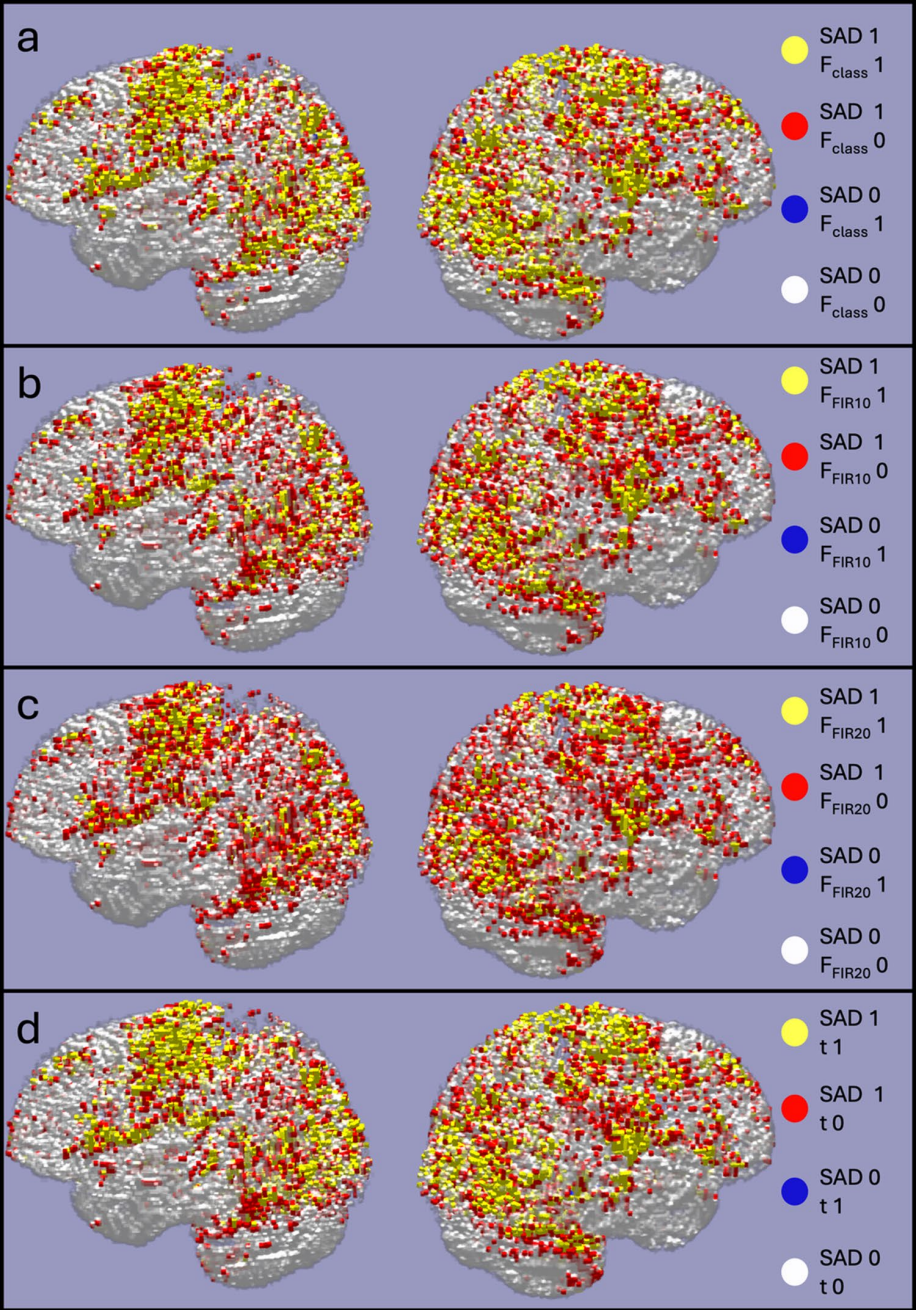
Color-coded 3D rendered plots comparing SAD classification with GLM statistics. **a** Spatial 3D rendered plots (left and right brain hemisphere) of all voxels in S_Test_ that were predicted active by SAD and/or F_class_. **b**–**d** Similar kind of visualization but for the comparisons between SAD and FIR_10_, FIR_20_ and t test statistics. In a large set of voxels, the SAD method agrees with each of the HRF model fits both when detecting activations (yellow) or deciding that the voxel time-series is a baseline (white). The SAD method also identifies many voxels that the HRF fits miss (red) but there are very few voxels where the opposite occurs (blue)

For completeness, the spatial distribution of all the voxels in S_Test_ that were predicted active by SAD and/or GLM, are shown in Fig. 8 as a 3D rendered plot.

**Fig. 9:**
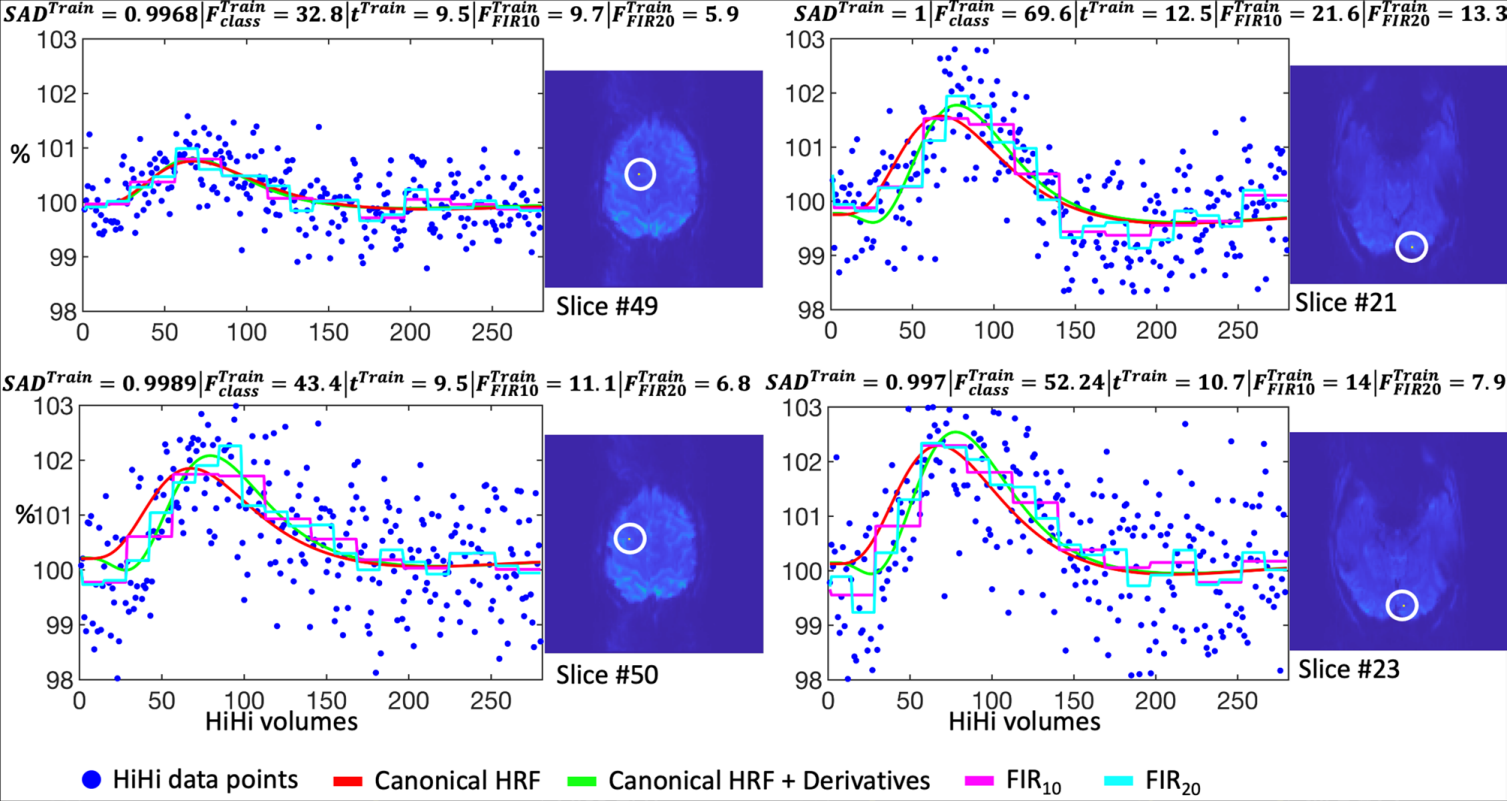
Comparison of GLM and SAD results. Left column: two examples of sampled activated time-series in the motor cortex sampled at 75-ms temporal resolution (blue dots) and its fitted responses using the canonical HRF alone (red line), together with its derivatives (green line), and a finite impulse response model with 10 (pink line) and 20 (cyan line) boxcar functions. The inset brain slice indicates the spatial location (yellow dot, white encircled) of the voxel. The title of each plotted time-series provides the corresponding predicted probability of SAD and the GLM test statistics values (F_Class_, t, F_FIR10_, and F_FIR20_). Right column: similar kind of plots but from the visual cortex. Note: that all time-series are scaled in [%] with respect to the mean time-course of the same voxel in the baseline dataset

Several BOLD responses sampled every 75-ms in S_Train_ dataset are provided from different anatomical locations in Fig. 9 along with their fitted responses (solid lines), SAD predictions and GLM test-statistics. The dependence of the GLM approach on the effect size is evident. While the SAD predictions are similar and close to 1 for effect sizes between ~ 0.7% and ~ 2.3%, the *t* and F statistics on the canonical (+ derivatives) or FIR HRF fits vary widely (even doubling in some cases).

**Fig. 10:**
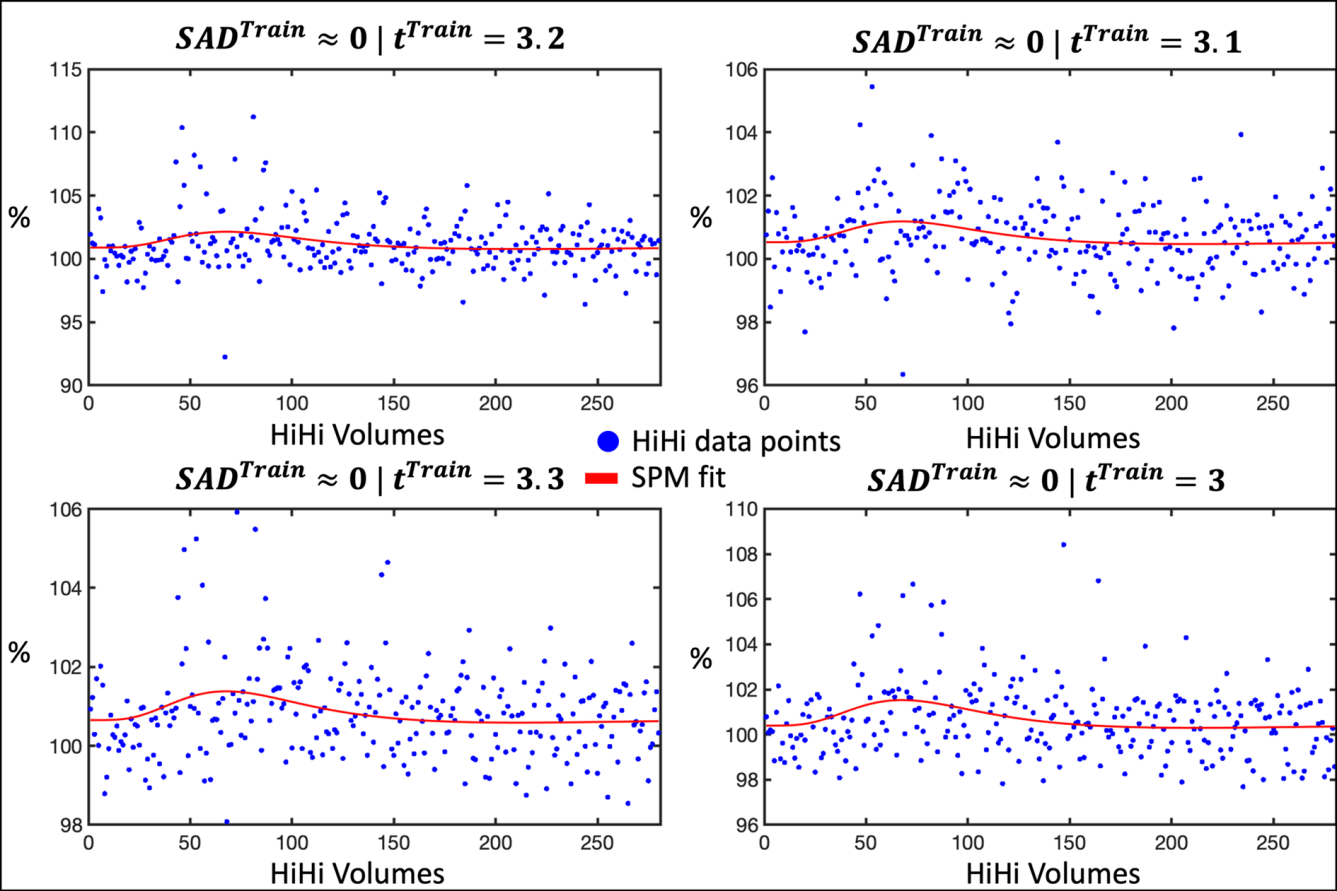
Example time-series with low SAD values but rather high t-test statistics. Illustration of different time-series from S_Train_ (blue dots) together with their fitted responses (red line) computed using the canonical HRF in SPM [17]. Notably, while the t statistic indicates a modest BOLD activation, the SAD method confidently predicts the contrary outcome (no BOLD response)

While Fig. 9 illustrated the superior sensitivity of the SAD method (in that it identified BOLD activations robustly even with a small effect size), Fig. 10 concerns its specificity. Examples are given, where a few large outliers around the expected timing of the peak of a BOLD response manifest as a small haemodynamic response when fitted with a canonical HRF model. Meanwhile the SAD predictions classify these time-series unequivocally as a baseline (no BOLD activation). Note that these kind of outlier time-series were not explicitly found in the 12′000 labelled voxel time-series in S_Test_.

Table 2 shows the results of the classification performance of the original data with 75-ms temporal resolution as well as several, progressively down-sampled versions. As expected, a decrease in temporal resolution is accompanied with a reduction in classification performance. Note that the AUC value does not necessarily mean high model performance. It is more an indication of the high discrimination index, i.e., the predicted probabilities for samples belonging to $C_{1}$ and $C_{0}$ exhibit significant divergence [41].

**Table 2:** Classification performance scores at different temporal resolutions

Resolution	TPR	TNR	PPV	ACC	F1	FNR	FPR	FDR	FOR	AUC	Iter Nr
75 ms	0.961 ± 0.009	0.998 ± 0.002	0.998 ± 0.002	0.998 ± 0.005	0.979 ± 0.005	0.038 ± 0.009	0.002 ± 0.002	0.002 ± 0.002	0.037 ± 0.009	0.998 ± 0.002	5
150 ms	0.904 ± 0.014	0.998 ± 0.001	0.998 ± 0.002	0.951 ± 0.002	0.949 ± 0.008	0.096 ± 0.014	0.001 ± 0.001	0.001 ± 0.002	0.088 ± 0.01	0.998 ± 0.001	3
225 ms	0.793 ± 0.017	0.999 ± 0.001	0.999 ± 0.002	0.896 ± 0.008	0.884 ± 0.01	0.207 ± 0.017	0.001 ± 0.001	0.001 ± 0.002	0.171 ± 0.012	0.984 ± 0.003	7
375 ms	0.705 ± 0.019	0.98 ± 0.006	0.973 ± 0.008	0.843 ± 0.01	0.818 ± 0.014	0.295 ± 0.019	0.02 ± 0.006	0.027 ± 0.008	0.231 ± 0.012	0.935 ± 0.008	6
750 ms	0.662 ± 0.019	0.905 ± 0.013	0.875 ± 0.015	0.784 ± 0.011	0.754 ± 0.014	0.338 ± 0.019	0.095 ± 0.013	0.125 ± 0.015	0.272 ± 0.011	0.835 ± 0.012	7

*TPR* true-positive rate, *TNR* true-negative rate, *PPV* positive-predictive value, *ACC* accuracy, *F1* harmonic mean of precision and sensitivity, *FNR* False-negative rate, *FPR* false-positive rate, *FDR* false discovery rate, *FOR* false-omission rate, *AUC* area under the receiver operating curve, *Iter Nr.* semi-supervised iteration number

## Discussion

We introduced a semi-supervised automatic detection (SAD) method for classifying voxels into two groups—those with or without a BOLD activation. The method is based on a Bi-LSTM neural network where initial labels are assigned based on the sample autocorrelation of the voxel time-series then undergoing iterative semi-supervised training. Classification performance of the trained model was assessed on an independent dataset from the same participant and showed excellent results. Moreover, in our evaluation contrasting SAD with conventional generalized linear models (GLM), we noted diminished reliance on the effect size and shape of specific hemodynamic responses both in the training dataset S_Train_ and the test set S_Test_. These combined advantages favor the proposed SAD method, particularly in relation to standard GLM statistics, when identifying activated time-series at the 1st statistical analysis level.

Various machine learning methods have been applied to fMRI data [29] and much of the field moved away from simply identifying the loci of activity—instead investigating multi-voxel patterns and how these patterns change across patient groups or as the stimulus or task is modulated. However, even these efforts often rely on a prior 1st level analysis, through which the areas of interest are identified. If machine learning provides a more reliable 1st level detection of voxel-wise BOLD activation, it would be beneficial for all subsequent advanced analyses. Although further work is needed on larger datasets, these findings suggest that the SAD method correctly identifies the manually labelled voxels with BOLD activations or baseline time-series (Fig. 6).

The decision to employ a relatively compact neural network with modest hidden dimensions stems from the balance between the available training data (≈40,000 voxels in the gray matter segment of a human brain) and the number of parameters (4734), which roughly adheres to the heuristic “10-times” rule of thumb in machine learning (see for instance: https://ts2.com.pl/en/what-is-the-10-times-rule-for-machine-learning/).

In Fig. 6, we compared the SAD performance to that of several conventionally used fitting methods, in particular to examine false positives/negatives—where one of the methods fails compared to the other. Of course, as it is common in machine learning, false positives/negatives are evaluated relative to the manual labelling and labelling a voxel as a baseline or activation is in the eye of the beholder. It is the opinion of the authors here, that a voxel time-series should be classified as ‘activated’ if it deviates consistently from a baseline random signal, regardless of the effect size or shape of the signal. For example, in the comparison between SAD vs F_Class_ in Fig. 6 (upper left), 1700 voxels were classified as an activation by SAD (~ 5% of those having a negative *t* value) but did not reach the threshold in the F_Class_ evaluation. On average, these voxel time-series contain an undeniable deviation from what one would expect as baseline. On the other hand, 22 voxels were suprathreshold in the F_Class_ evaluation while classified as baseline by SAD—although half of these voxels contained a negative activation (and negative *t* value). Supplementary Fig. 2a compares these positive and negative activations. As can be seen in Supplementary Fig. 2b, the shape of such activations can be ambiguous—it is difficult to decide by eye whether it’s a negative activation with a large “initial dip” or a positive activation with an early rise and large undershoot. Nevertheless, if the neural network model does not see examples of such voxel time-series in the training phase, it will struggle to classify it correctly.

Another reason why a model could struggle is when it sees and it is trained on lower quality data. For this reason, we investigated the effect of lower temporal resolution on SAD performance by downsampling the original 75-ms dataset. Unsurprisingly, classification performance deteriorated as the sampling rate was reduced. Although some success was still achieved at sampling times of ~ 200-ms the SAD method is not applicable to conventional data where the TR is 1–3 s. This is due mainly to the reliance of the initialization step on the autocorrelation of the time-series, which is poorly estimated when there are only 7–20 samples in the 20 s window of a BOLD response. Additionally, as is shown in Supplementary Fig. 1, distribution of the sum of autocorrelations becomes symmetric and narrow—i.e. with 7–20 samples baseline and BOLD responses are less distinguishable based on their autocorrelation and thus do not provide proper initial separation between the $C_{0}$ and $C_{1}$.

The BOLD response is usually a slowly varying signal, thus theoretically, sampling rates of double the highest frequency in the BOLD response should suffice for distinguishing activated and baseline time-series. However, recent evidence indicates that despite its seemingly sluggish evolution, the BOLD signal can encode high frequency stimuli [22, 42]. To identify and extract this modulation in the BOLD signal, high frequency sampling is needed. When such modulation still results in smooth, slowly varying responses (e.g., changing the timing of the peak or the slope of the initial dip) the SAD method can suitably be applied. However, because the SAD method relies on high temporal autocorrelation, detecting BOLD responses with high-frequency oscillations would be more difficult and would have to be trained specifically (e.g., reducing the autocorrelation lag from 19 to 10). Nevertheless, rapid temporal sampling is beneficial to detect both small temporal changes in the overall smooth shape and capturing high-frequency content, and the rapid sampling improves also the initialization of the SAD procedure via the sample autocorrelation function.

Neural network models perform best if they are trained in a supervised fashion. However, labelling voxels is laborious and the BOLD response can vary within and between individuals especially in disease [43, 44]. For this reason, the ideal approach would be unsupervised clustering of the voxels in every dataset without prior knowledge of the mental or disease state of the individual scanned. However, unsupervised learning strategies remain very challenging due to imprecise objectives (e.g., prediction error and evaluation performance) that amplifies ambiguity and further complicates the interpretation of the results. These constraints led us to the development of the present semi-supervised approach, where only a few labels need to be automatically assigned initially. Further labels are created through subsequent training in an iterative unsupervised manner.

Figure 10 illustrates how easily the conventional t test can be ‘fooled’ by a few outliers. Although, the resulting t statistic did not reach the threshold here, investigators often report results without correction for multiple comparisons where some of these false positive activations can sneak through. Despite the sensitivity and specificity of the SAD method (Table 2), further work is needed in properly treating multiple comparisons.

The present work was a proof of principle effort. Other future developments may examine the generalizability of the trained model: i.e., whether it is sufficient to train on a single participant and apply it to the entire study on the one extreme or whether each participant will need their own model in the other extreme.

## Conclusion

The semi-supervised automatic detection (SAD) method, which is based on a Bi-LSTM neural network, detected hemodynamic responses (activated time-series) with different effect sizes in the human brain at various temporal resolutions but performed better at higher temporal resolutions ≤ 375-ms. SAD is presently applicable to HiHi fMRI time-series, and there is potential for future work to broaden its applicability to include a more extensive participant pool and diverse fMRI paradigms.

## Supplementary Information

Below is the link to the electronic supplementary material.

Supplementary file1: Figure 1. (**a**-**e**) Histograms of for the corresponding temporal resolution, after downsampling the original (75-ms) fMRI time-series. The blue and red solid lines represent thresholds for the initial class assignment. With decreasing resolution, the histogram becomes less skewed and more narrow, resulting in a reduced difference between the two classes (blue and red line are getting closer). Thus, with decreasing temporal resolution the initial labels are less unique in the two classes of the training pool, which can lead to worse classification performance. (**f**) Assembly of hyperparameters () for each downsampled temporal resolution that were used/computed at the training stage. Note that was computed with Eq. (4) but inherently depends on (TIFF 15685 KB)

Supplementary file2: Figure 2. (**a**) (Top row) Mean time-course (black dots) of 11 voxels with negative t-values where SAD is below 0.5 but FClass is above the p = 0.05 FWER threshold together with its fitted response using the canonical HRF with (green solid line) and without (red solid line) its derivatives. Only one of those 11 time-series has an absolute t-value greater than 5.4 (p = 0.05 FWER threshold). Note that the y-axis is scaled in [%] with respect to the mean time-course of the corresponding voxels in the baseline time-series. (Bottom row) Same kind of plot corresponding to the other 11 voxel time-series with a positive t-value where SAD is below 0.5 but FClass is above the p = 0.05 FWER threshold. (**b**) Example time-course of the 182 manually labelled negative sampled BOLD responses (black dots) together with its canonical fit in SPM (red solid line). If the time-course is truly stemming from a slow negative or a fast (~3s) positive BOLD response is subjective. However, the data indicate a distinct deviation from the canonical HRF which confounds the statistical result obtained through the t-test (t $\approx$ 2.09) (TIFF 20441 KB)

Supplementary file3: Figure 3. **Scatter plots and GLM fits for all combinations of positive/negative SAD classification probabilities and F/t-statistics from** **S**_**Test**_. The insets in Fig. 6 provided the scatter plots for voxels in which the time series we classified as baseline by the GLM statistics but as an activation by SAD. In this supplementary figure we provide all the other permutations of activation/baseline as detected by two methods at a time (TIFF 23989 KB)

Supplementary file4: Figure 4. **HiHi in a Nutshell**. The parallelograms represent the individual EPI slices in an fMRI experiment acquired with 20 slices and a 1.5s (long) TR. The RF-excitations of slice 1 in EPI volume 1 and slice 2 in EPI volume 15 (plain and checkered red slices) temporally coincide with the first and second stimulus (red arrows), respectively. The time between two stimuli (14 x 1.5s = 21s) allows the BOLD response to play out fully before returning to the baseline fMRI signal. The slices after a stimulus (e.g. slice 2, 3 and 20 are color-coded blue, green and violet, respectively) are mapped to the same slice in the HiHi time series, but shifted in time in relation to the slice timing of the stimulus. This way, the slice TR (here 1.5s/20slices = 0.075s) becomes the effective volume TR of the HiHi time-series. Any voxel time-series that show a BOLD activation in the HiHi time-series (yellow square in the HiHi time-series) can then either be found manually, by using SAD or a standard GLM approach (TIFF 34719 KB)

## Data Availability

The data and code used in this study are available from the authors upon reasonable request.

## References

[1] Deyoe EA, Bandettini P, Neitz J, Miller D, Winans P (1994) Functional magnetic resonance imaging (FMRI) of the human brain. J Neurosci Methods. 10.1016/0165-0270(94)90191-0 7869750 10.1016/0165-0270(94)90191-0

[2] Magistretti PJ, Pellerin L (1999) Cellular mechanisms of brain energy metabolism and their relevance to functional brain imaging. Phil Trans: Biol Sci 354:1155–1163 10.1098/rstb.1999.0471 PMC1692634 10466143

[3] Buxton RB, Uludaǧ K, Dubowitz DJ, Liu TT (2004) Modeling the hemodynamic response to brain activation. Neuroimage. 10.1016/j.neuroimage.2004.07.013 15501093 10.1016/j.neuroimage.2004.07.013

[4] Ogawa S, Lee TM, Kay AR, Tank DW (1990) Brain magnetic resonance imaging with contrast dependent on blood oxygenation (cerebral blood flow/brain metabolism/oxygenation). Proc Natl Acad Sci U S A 87:9868–9872 2124706 10.1073/pnas.87.24.9868 PMC55275

[5] Turner R, Le BD, Moonen CTW, Despres D, Frank J (1991) Echo-planar time course MRI of cat brain oxygenation changes. Magn Reson Med 22:159–166 1798390 10.1002/mrm.1910220117

[6] Kwong KK, Belliveaut JW, Cheslert DA, Goldbergt IE, Weisskofft RM, Poncelett BP, Kennedyt DN, Hoppelt BE, Cohent MS, Turnert R, Cheng H-M, Bradyt TJ, Rosent BR (1992) Dynamic magnetic resonance imaging of human brain activity during primary sensory stimulation. Proc Natl Acad Sci U S A 89:5675–5679 1608978 10.1073/pnas.89.12.5675 PMC49355

[7] Bandettini PA, Wong EC, Hinks RS, Tikofsky RS, Hyde JS (1992) Time course EPI of human brain function during task activation. Magn Reson Med 25:390–397 1614324 10.1002/mrm.1910250220

[8] Pauling L, Coryell CD (1936) The magnetic properties and structure of hemoglobin, oxyhemoglobin and carbonmonoxyhemoglobin. Proc Natl Acad Sci U S A 22:210–216 16577697 10.1073/pnas.22.4.210 PMC1076743

[9] Detre JA, Wang J (2002) Technical aspects and utility of fMRI using BOLD and ASL. Clin Neurophysiol. 113(5):621–634. 10.1016/S1388-2457(02)00038-X 11976042 10.1016/s1388-2457(02)00038-x

[10] Logothetis NK, Pfeuffer J (2004) On the nature of the BOLD fMRI contrast mechanism. Magn Reson Imaging. Elsevier Inc., pp 1517–1531 10.1016/j.mri.2004.10.018 15707801

[11] Monti MM (2011) Statistical analysis of fMRI time-series: a critical review of the GLM approach. Front Hum Neurosci. 10.3389/fnhum.2011.00028 21442013 10.3389/fnhum.2011.00028 PMC3062970

[12] Friston KJ, Josephs O, Zarahn E, Holmes AP, Rouquette S, Poline JB (2000) To smooth or not to smooth? Bias and efficiency in fMRI time-series analysis. Neuroimage 12:196–208 10913325 10.1006/nimg.2000.0609

[13] Logothetis NK (2008) What we can do and what we cannot do with fMRI. Nature 453:869–878 18548064 10.1038/nature06976

[14] Soares JF, Abreu R, Lima AC, Sousa L, Batista S, Castelo-Branco M, Duarte JV (2022) Task-based functional MRI challenges in clinical neuroscience: choice of the best head motion correction approach in multiple sclerosis. Front Neurosci. 10.3389/fnins.2022.1017211 36570849 10.3389/fnins.2022.1017211 PMC9768441

[15] Elliott ML, Knodt AR, Ireland D, Morris ML, Poulton R, Ramrakha S, Sison ML, Moffitt TE, Caspi A, Hariri AR (2020) What is the test-retest reliability of common task-functional MRI measures? New empirical evidence and a meta-analysis. Psychol Sci 31:792–806 32489141 10.1177/0956797620916786 PMC7370246

[16] Bennett CM, Miller MB (2010) How reliable are the results from functional magnetic resonance imaging? Ann N Y Acad Sci 1191:133–155 20392279 10.1111/j.1749-6632.2010.05446.x

[17] Friston KJ, Ashburner JT, Kiebel SJ, Nichols TE, Penny William D (2007) Statistical parametric mapping: the analysis of functional brain images, 1st edn. Academic Press

[18] McGonigle DJ, Howseman AM, Athwal BS, Friston KJ, Frackowiak RSJ, Holmes AP (2000) Variability in fMRI: an examination of intersession differences. Neuroimage 11:708–734 10860798 10.1006/nimg.2000.0562

[19] Handwerker DA, Gonzalez-Castillo J, D’Esposito M, Bandettini PA (2012) The continuing challenge of understanding and modeling hemodynamic variation in fMRI. Neuroimage 62:1017–1023 22366081 10.1016/j.neuroimage.2012.02.015 PMC4180210

[20] Gonzalez-Castillo J, Saad ZS, Handwerker DA, Inati SJ, Brenowitz N, Bandettini PA (2012) Whole-brain, time-locked activation with simple tasks revealed using massive averaging and model-free analysis. Proc Natl Acad Sci U S A 109:5487–5492 22431587 10.1073/pnas.1121049109 PMC3325687

[21] Handwerker DA, Ollinger JM, D’Esposito M (2004) Variation of BOLD hemodynamic responses across subjects and brain regions and their effects on statistical analyses. Neuroimage 21:1639–1651 15050587 10.1016/j.neuroimage.2003.11.029

[22] Polimeni JR, Lewis LD (2021) Imaging faster neural dynamics with fast fMRI: a need for updated models of the hemodynamic response. Prog Neurobiol. 10.1016/j.pneurobio.2021.102174 34525404 10.1016/j.pneurobio.2021.102174 PMC8688322

[23] Larkman DJ, Hajnal JV, Herlihy AH, Coutts GA, Young IR, Sta Ehnholm G (2001) Use of multicoil arrays for separation of signal from multiple slices simultaneously excited. Magn Reson Imaging 13:313–317 10.1002/1522-2586(200102)13:2<313::aid-jmri1045>3.0.co;2-w 11169840

[24] Barth M, Breuer F, Koopmans PJ, Norris DG, Poser BA (2016) Simultaneous multislice (SMS) imaging techniques. Magn Reson Med 75:63–81 26308571 10.1002/mrm.25897 PMC4915494

[25] Moeller S, Yacoub E, Olman CA, Auerbach E, Strupp J, Harel N, Uǧurbil K (2010) Multiband multislice GE-EPI at 7 tesla, with 16-fold acceleration using partial parallel imaging with application to high spatial and temporal whole-brain FMRI. Magn Reson Med 63:1144–1153 20432285 10.1002/mrm.22361 PMC2906244

[26] Schmidt T, Vannesjo SJ, Sommer S, Nagy Z (2023) fMRI with whole-brain coverage, 75-ms temporal resolution and high SNR by combining HiHi reshuffling and multiband imaging. Magn Reson Imaging. 10.1016/j.mri.2023.06.015 37385353 10.1016/j.mri.2023.06.015

[27] Nagy Z, Hutton C, David G, Hinterholzer N, Deichmann R, Weiskopf N, Vannesjo SJ (2022) HiHi fMRI: a data-reordering method for measuring the hemodynamic response of the brain with high temporal resolution and high SNR. Cereb Cortex. 10.1093/cercor/bhac364 10.1093/cercor/bhac364 PMC10110425 36169574

[28] Bishop MC (2006) Pattern recognition and machine learning, 1st edn. Springer, Cambridge

[29] Pereira F, Mitchell T, Botvinick M (2009) Machine learning classifiers and fMRI: a tutorial overview. Neuroimage. 10.1016/j.neuroimage.2008.11.007 19070668 10.1016/j.neuroimage.2008.11.007 PMC2892746

[30] Wen D, Wei Z, Zhou Y, Li G, Zhang X, Han W (2018) Deep learning methods to process fMRI data and their application in the diagnosis of cognitive impairment: a brief overview and our opinion. Front Neuroinform. 10.3389/fninf.2018.00023 29755334 10.3389/fninf.2018.00023 PMC5932168

[31] Dvornek NC, Ventola P, Pelphrey KA, Duncan JS (2017) Identifying autism from resting-state fMRI using long short-term memory networks. Lecture Notes in Computer Science (including subseries Lecture Notes in Artificial Intelligence and Lecture Notes in Bioinformatics). Springer Verlag, Cham, pp 362–370 10.1007/978-3-319-67389-9_42 PMC5669262 29104967

[32] Hochreiter S, Schmidhuber J (1997) Long short-term memory. Neural Comput 9:1735–1780 9377276 10.1162/neco.1997.9.8.1735

[33] Li H, Fan Y (2019) Interpretable, highly accurate brain decoding of subtly distinct brain states from functional MRI using intrinsic functional networks and long short-term memory recurrent neural networks. Neuroimage. 10.1016/j.neuroimage.2019.116059 31362049 10.1016/j.neuroimage.2019.116059 PMC6819260

[34] Brockwell PJ, Davis RA (2016) Springer Texts in Statistics Introduction to Time Series and Forecasting, 3rd edn. Springer Nature, Switzerland

[35] Gatto R (2020) Stochastische Modelle der aktuariellen Risikotheorie Eine mathematische Einführung, 2nd edn. Masterclass, Heidelberg

[36] Ioffe S, Szegedy C (2015) Batch normalization: accelerating deep network training by reducing internal covariate shift. arXiv. 10.48550/arXiv.1502.03167

[37] Lee D-H Pseudo-Label (2013) The simple and efficient semi-supervised learning method for deep neural networks. ICML 2013 Workshop: Challenges in Representation Learning (WREPL), Atlanta, Georgia, USA

[38] Kingma DP, Ba J (2015) Adam: a method for stochastic optimization. In: International conference on learning representations, San Diego, USA

[39] Schmitt F, Stehling MK, Turner R (1998) Echo-planar imaging, 1st edn. Springer, Berlin Heidelberg

[40] Marques JP, Kober T, Krueger G, van der Zwaag W, Van de Moortele PF, Gruetter R (2010) MP2RAGE, a self bias-field corrected sequence for improved segmentation and T1-mapping at high field. Neuroimage 49:1271–1281 19819338 10.1016/j.neuroimage.2009.10.002

[41] Lobo JM, Jiménez-valverde A, Real R (2008) AUC: a misleading measure of the performance of predictive distribution models. Glob Ecol Biogeogr 17:145–151

[42] Lewis LD, Setsompop K, Rosen BR, Polimeni JR (2016) Fast fMRI can detect oscillatory neural activity in humans. Proc Natl Acad Sci U S A 113:E6679–E6685 27729529 10.1073/pnas.1608117113 PMC5087037

[43] Bonakdarpour B, Parrish TB, Thompson CK (2007) Hemodynamic response function in patients with stroke-induced aphasia: implications for fMRI data analysis. Neuroimage 36:322–331 17467297 10.1016/j.neuroimage.2007.02.035 PMC2041913

[44] Duarte JV, Guerra C, Moreno C, Gomes L, Castelo-Branco M (2023) Changes in hemodynamic response function components reveal specific changes in neurovascular coupling in type 2 diabetes. Front Physiol. 10.3389/fphys.2022.1101470 36703928 10.3389/fphys.2022.1101470 PMC9872943

