# Supplementary figures and images for: SAD: semi-supervised automatic detection of BOLD activations in high temporal resolution fMRI data
Source: MAGMA. 2024 Aug 29;37(6):1031–46. doi: 10.1007/s10334-024-01197-0 (PMC11582144; doi:10.1007/s10334-024-01197-0)

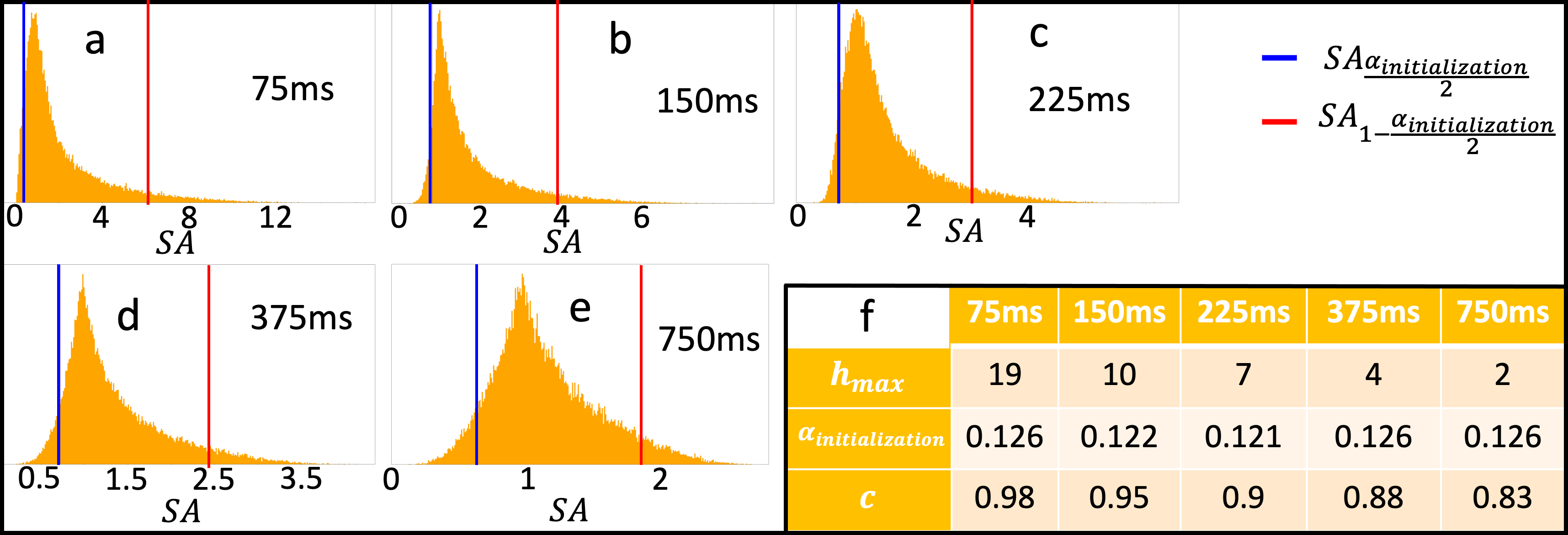

Supplement: Supplementary file 1 — Supplementary file1: Figure 1. (a-e) Histograms of for the corresponding temporal resolution, after downsampling the original (75-ms) fMRI time-series. The blue and red solid lines represent thresholds for the initial class assignment. With decreasing resolution, the histogram becomes less skewed and more narrow, resulting in a reduced difference between the two classes (blue and red line are getting closer). Thus, with decreasing temporal resolution the initial labels are less unique in the two classes of the training pool, which can lead to worse classification performance. (f) Assembly of hyperparameters () for each downsampled temporal resolution that were used/computed at the training stage. Note that was computed with Eq. (4) but inherently depends on (TIFF 15685 KB) [file 10334_2024_1197_MOESM1_ESM.tiff]

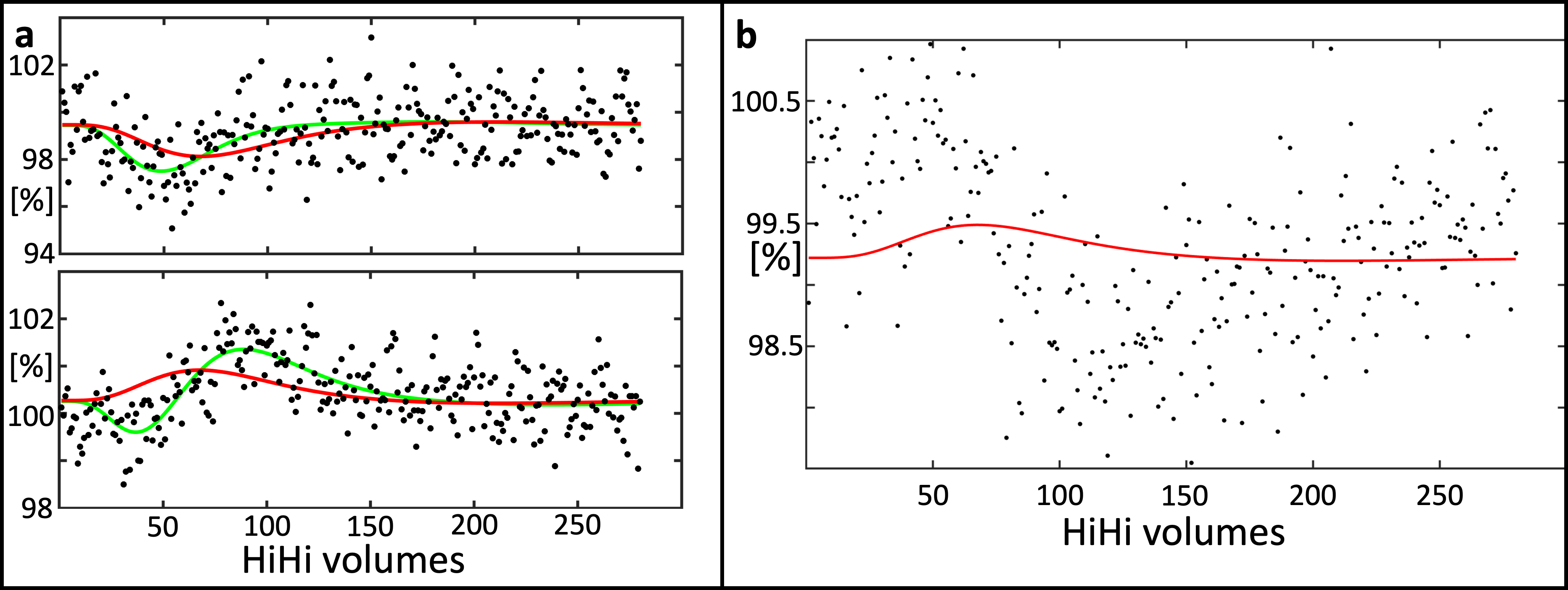

Supplement: Supplementary file 2 — Supplementary file2: Figure 2. (a) (Top row) Mean time-course (black dots) of 11 voxels with negative t-values where SAD is below 0.5 but FClass is above the p = 0.05 FWER threshold together with its fitted response using the canonical HRF with (green solid line) and without (red solid line) its derivatives. Only one of those 11 time-series has an absolute t-value greater than 5.4 (p = 0.05 FWER threshold). Note that the y-axis is scaled in [%] with respect to the mean time-course of the corresponding voxels in the baseline time-series. (Bottom row) Same kind of plot corresponding to the other 11 voxel time-series with a positive t-value where SAD is below 0.5 but FClass is above the p = 0.05 FWER threshold. (b) Example time-course of the 182 manually labelled negative sampled BOLD responses (black dots) together with its canonical fit in SPM (red solid line). If the time-course is truly stemming from a slow negative or a fast (~3s) positive BOLD response is subjective. However, the data indicate a distinct deviation from the canonical HRF which confounds the statistical result obtained through the t-test (t $\approx$≈ 2.09) (TIFF 20441 KB) [file 10334_2024_1197_MOESM2_ESM.tiff]

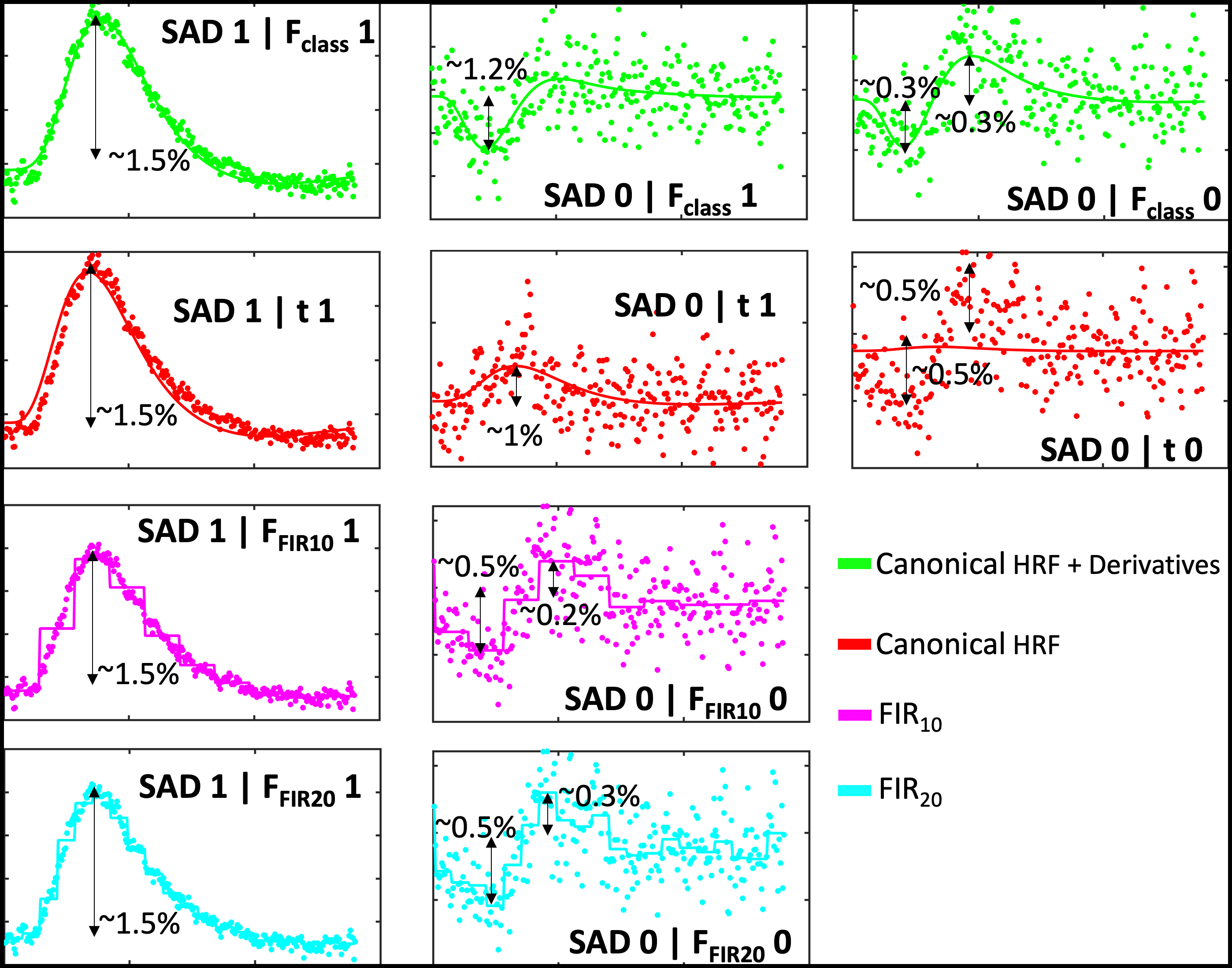

Supplement: Supplementary file 3 — Supplementary file3: Figure 3. Scatter plots and GLM fits for all combinations of positive/negative SAD classification probabilities and F/t-statistics from STest. The insets in Fig. 6 provided the scatter plots for voxels in which the time series we classified as baseline by the GLM statistics but as an activation by SAD. In this supplementary figure we provide all the other permutations of activation/baseline as detected by two methods at a time (TIFF 23989 KB) [file 10334_2024_1197_MOESM3_ESM.tiff]

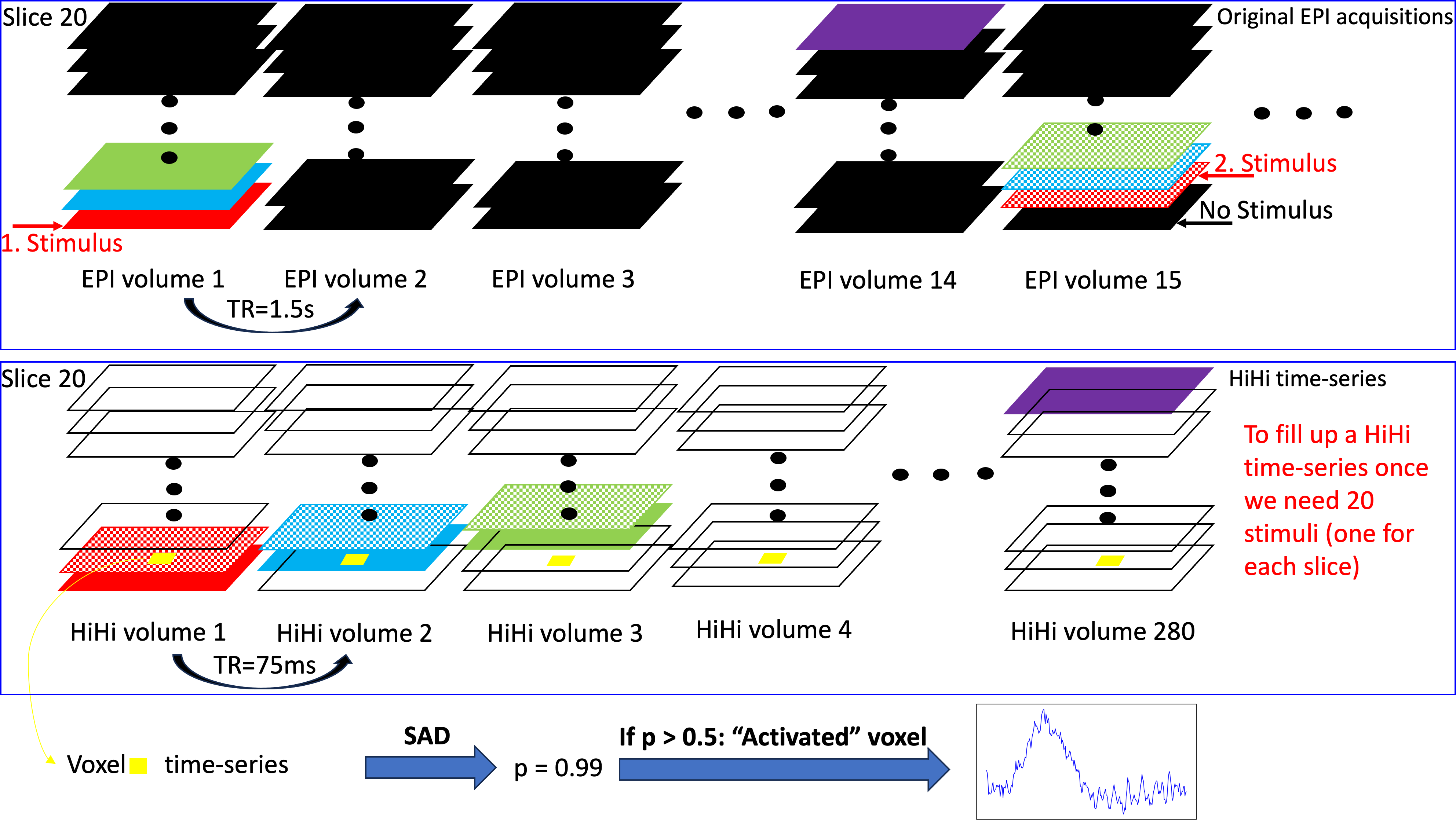

Supplement: Supplementary file 4 — Supplementary file4: Figure 4. HiHi in a Nutshell. The parallelograms represent the individual EPI slices in an fMRI experiment acquired with 20 slices and a 1.5s (long) TR. The RF-excitations of slice 1 in EPI volume 1 and slice 2 in EPI volume 15 (plain and checkered red slices) temporally coincide with the first and second stimulus (red arrows), respectively. The time between two stimuli (14 x 1.5s = 21s) allows the BOLD response to play out fully before returning to the baseline fMRI signal. The slices after a stimulus (e.g. slice 2, 3 and 20 are color-coded blue, green and violet, respectively) are mapped to the same slice in the HiHi time series, but shifted in time in relation to the slice timing of the stimulus. This way, the slice TR (here 1.5s/20slices = 0.075s) becomes the effective volume TR of the HiHi time-series. Any voxel time-series that show a BOLD activation in the HiHi time-series (yellow square in the HiHi time-series) can then either be found manually, by using SAD or a standard GLM approach (TIFF 34719 KB) [file 10334_2024_1197_MOESM4_ESM.tiff]
